# Dissecting Tertiary Lymphoid Structures in Cancer: Maturation, Localization and Density

**DOI:** 10.7150/thno.113940

**Published:** 2025-08-30

**Authors:** Guang-Liang Su, Meng-Jie Zhang, Hao Li, Zhi-Jun Sun

**Affiliations:** 1 State Key Laboratory of Oral & Maxillofacial Reconstruction and Regeneration, Key Laboratory of Oral Biomedicine Ministry of Education, Hubei Key Laboratory of Stomatology, School & Hospital of Stomatology, Frontier Science Center for Immunology and Metabolism, Taikang Center for Life and Medical Sciences, Wuhan University, Wuhan 430079, China.; 2 Department of Oral Maxillofacial-Head Neck Oncology, School & Hospital of Stomatology, Wuhan University, Wuhan 430079, China.

**Keywords:** Tertiary lymphoid structures, Heterogeneity, Maturation, Localization, Density

## Abstract

Tertiary lymphoid structures (TLSs) refer to ectopic lymphoid aggregates that form in non-lymphoid tissues at sites of chronic inflammation including cancers. TLSs have been recognized as significant predictors of the efficacy of immune checkpoint blockade (ICB) therapies and have the potential to elicit robust anti-tumor immune response. However, recent studies have revealed substantial heterogeneity in TLSs across different individuals and cancer types, which directly impacts the effectiveness of anti-tumor immunity. Concretely, the maturation status, localization, and density of TLSs profoundly influence the dynamic interactions among immune cells within these structures, potentially leading to adverse effects. This review provides an in-depth exploration of how the heterogeneity of TLSs influences cellular composition and immune dynamics, with the objective of influencing the efficacy of ICB therapies and modulating prognostic prediction accuracy. Additionally, the potential of combining TLSs with other biomarkers for predicting anti-tumor immunity outcomes is further investigated, alongside the introduction of advanced technologies for evaluating TLS heterogeneity. Collectively, these analyses aim to advance the understanding of TLS heterogeneity and facilitate its translation into clinical and translational medicine applications.

## Introduction

Immune checkpoint blockade (ICB) therapy has unleashed anti-tumor immune response, leading to unprecedented durable response rates in various types of cancer 1. However, due to primary and acquired resistance as well as toxicity associated with ICB, the number of patients benefiting from this treatment remains limited 2. Consequently, there is an urgent need to develop diagnostic tools to identify patients who may benefit from ICB while seeking appropriate strategies to improve therapeutic outcomes. Programmed death ligand 1 (PD-L1) expression, microsatellite instability-high/defective mismatch repair (MSI-H/dMMR), and tumor mutation burden (TMB) are common predictive biomarkers in clinical practice 3-5. Nevertheless, these biomarkers alone cannot fully predict immunotherapy responsiveness, underscoring the need to identify more precise biomarkers for therapeutic efficacy evaluation. Recent studies found that tertiary lymphoid structures (TLSs) demonstrate robust potential in improving prognosis and enhancing response to ICB therapy and may serve as a significant source of anti-tumor immunity within solid tumors 6-9.

TLSs are ectopic lymphoid aggregates that form within non-lymphoid tissues, capable of locally generating tumor-specific effector T cells, B cells, and antibodies, playing a crucial role in establishing an anti-tumor immune environment 10-12. Current research indicates that the formation of TLSs is primarily driven by persistent antigenic stimulation within the chronic inflammatory microenvironment 13. This encompasses a variety of conditions, including autoimmune disorders, persistent infections, and particularly cancer 14. Under inflammation-induced conditions, lymphocytes accumulate and gradually organize into immune units similar to secondary lymphoid organs (SLOs), differentiating into B cell zones containing germinal centers (GCs) and surrounding T cell zones 15. While the triggers and processes involved in the origin and development of TLSs have become increasingly clear 16, 17, several unresolved questions remain regarding their role in predicting and enhancing anti-tumor immune effects. Concretely, TLSs exhibit notable heterogeneity across patients and cancer types, involving differences in maturation status, localization, and density 10, 11, 18. These variations significantly impact changes in the cellular composition and anti-tumor immunity of TLSs, which may lead to negative clinical prognosis 19, 20. Thus, a deeper understanding of the mechanisms underlying the formation of TLS heterogeneity and its impact on anti-tumor immunity is essential. Combined with improvements in evaluation strategies and technologies, it will enhance the accuracy of ICB prognostic predictions and immunotherapy effectiveness.

**Figure 1:**
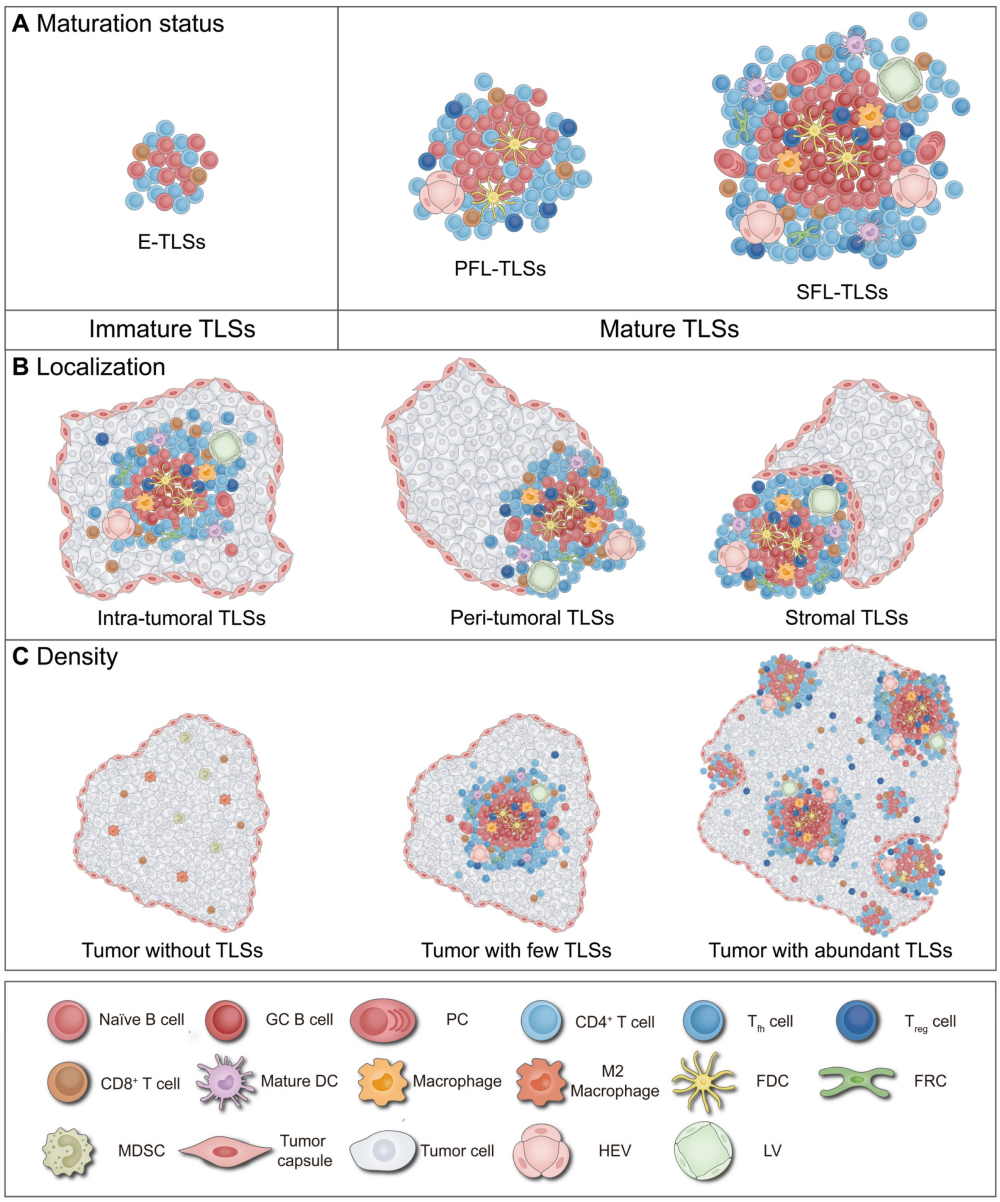
** Definition of TLS heterogeneity.** TLSs exhibit marked heterogeneity in maturation, localization and density across tumor types and individuals, profoundly impacting anti-tumor immune effects and clinical value.** (A) Maturation status of TLSs.** The maturation status of TLSs is classified into three categories: loosely aggregated lymphoid cells; primary follicles containing T cells, B cells, and FDCs; and mature polarized structures featuring GCs, HEVs, and a variety of immune cells, such as macrophages.** (B) Localization of TLSs.** TLSs are variably distributed within the body and can be categorized based on their localization as intra-tumoral, peri-tumoral, or within the tumor stroma. **(C) Density of TLSs.** The presence of TLSs indicates a robust anti-tumor immune response, capable of converting “cold” tumors into “hot” tumors. Moreover, higher density of TLSs are generally associated with better clinical outcomes. DC: dendritic cell; E-TLSs: early tertiary lymphoid structures; FDC: follicular dendritic cell; FRC: fibroblastic reticular cell; GC: germinal center; HEV: high endothelial venule; LV: lymphatic vessel; MDSC: myeloid-derived suppressor cell; NK: natural killer; PC: plasma cell; PFL-TLSs: primary follicle-like tertiary lymphoid structures; SFL-TLSs: secondary follicle-like tertiary lymphoid structures; T_fh_: T follicular helper; T_reg_: regulatory T cell.

This review summarizes the latest research on TLSs, focusing on immune dynamics in maturation, localization and density (Figure 1) 10, 11, 18-20. First, by integrating existing evidence, this paper systematically explains the definition and influencing factors of TLS heterogeneity, as well as its significance for tumor immunity and clinical practice. Second, it explores the future development of combining TLS heterogeneity evaluation with other biomarkers to predict anti-tumor immune outcomes, while summarizing current advanced methods for assessing TLS heterogeneity. Finally, this paper outlines key directions for future research, including optimizing evaluation strategies, improving assessment technologies, and developing advanced intervention materials, aiming to further refine and enhance clinical transformation and application.

### Structural characteristics and heterogeneity of TLSs

**Figure 2:**
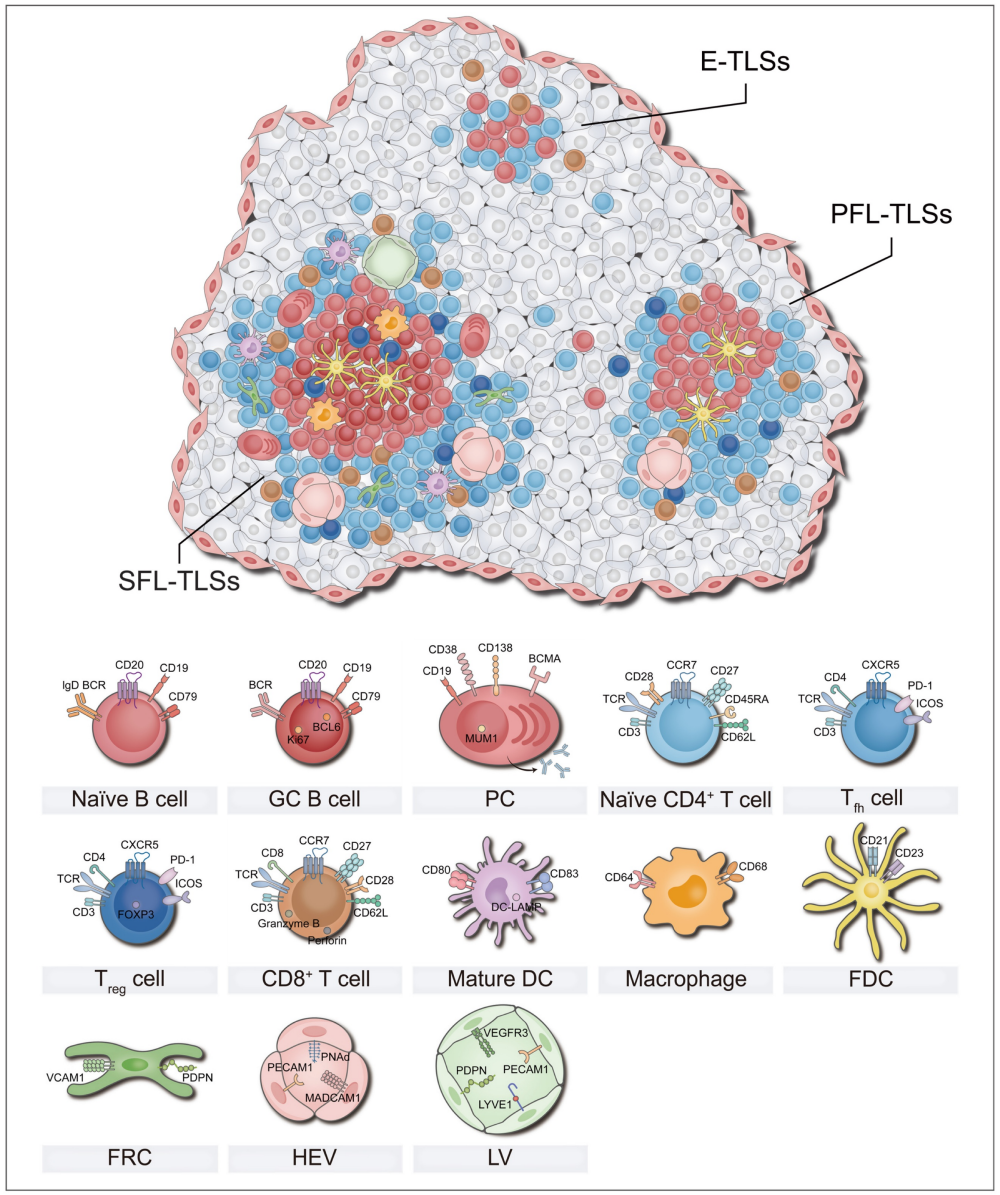
** Cellular composition of TLSs.** The cellular composition of TLSs varies under different mature status, which affects anti-tumor immunity. Mature TLSs are composed of a diverse array of cell types, including T cells, B cells, DCs, FDCs, FRCs, macrophages, and HEVs *et al.* These cells perform distinct roles, collectively establishing and maintaining an immune niche within the tumor microenvironment that is either anti-tumoral or pro-tumoral. DC: dendritic cell; E-TLSs: early tertiary lymphoid structures; FDC: follicular dendritic cell; FRC: fibroblastic reticular cell; GC: germinal center; HEV: high endothelial venule; LV: Lymphatic vessel; MDSC: myeloid-derived suppressor cell; PC: plasma cell; PFL-TLSs: primary follicle-like tertiary lymphoid structures; SFL-TLSs: secondary follicle-like tertiary lymphoid structures; T_fh_: T follicular helper; T_reg_: regulatory T cell.

TLSs are temporary immune unit formed in chronic inflammation, featuring distinct T and B cell zones at their core 11. The T cell region is dominated by CD4^+^ cells, which supports the activation of naïve B cells and the formation of GCs by facilitating antigen presentation 21. Additionally, dendritic cells (DCs) expressing dendritic cell-lysosomal-associated membrane protein (DC-LAMP, also called LAMP3) located in this region can capture, process, and present antigens to activate initial CD8^+^ T cells 22. In the TLSs, CD8^+^ T cells exert their anti-tumor immune effects by directly killing tumor cells and secreting effector molecules (*e.g.*, granzyme B) 18. CD20^+^ B cell follicles with GCs, supported by a network of follicular dendritic cells (FDCs), serve as vital sites for the proliferation of B cells and antibody class transformation 23. Moreover, CD21^+^ FDCs located in this region play a critical role in the selection of memory B cells during GC reactions 24. Besides serving as organized congregations of T and B cells, TLSs also encompass a diverse array of immune cells such as macrophages, natural killer (NK) cells, and neutrophils 23, 25, 26. In addition, PNAd^+^ high endothelial venules (HEVs) constitute the vascular system of TLSs, and recruit circulating lymphocytes into TLSs by secreting chemokines such as CC motif chemokine ligand 19 (CCL19) and CCL21, along with adhesion molecules 10. The dense stromal network constructed by fibroblastic reticular cells (FRCs) supports the entire TLS structure and anchors it at the inflamed tissue, ensuring effective local immune response 27. In short, T/B cell compartments, other immune cells, HEVs, and the matrix network collectively constitute a dynamic and highly organized anti-tumor immune microenvironment (Figure 2).

However, it is important to note that not all cancer patients possess TLSs, and not all TLSs exhibit complete structures 10, 11, 18. Clinical and translational evidence has demonstrated substantial heterogeneity in TLS presence across cancer types, molecular subtypes, and disease stages 28-31. Even when present, many TLSs display structural incompleteness—manifesting as lymphocyte aggregates lacking GCs or disorganized T/B cell compartmentalization 30, 32, 33. These structurally impaired TLSs show functional limitations: the absence of GCs disrupts B cell affinity maturation, ultimately weakening responses to immunotherapy and correlating with reduced patient survival 6-9.

### Formation and development of TLSs

**Figure 3:**
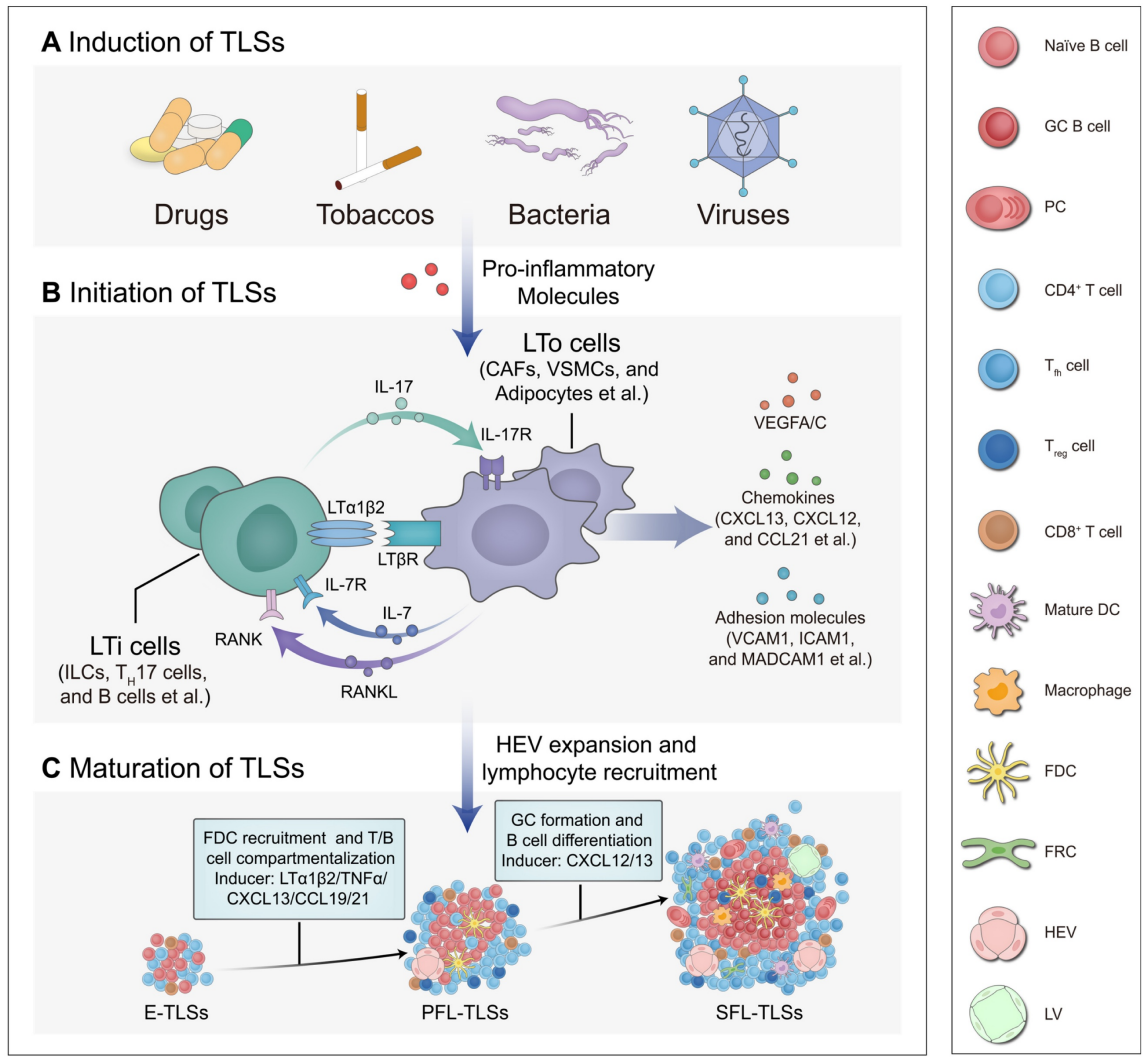
** Development of TLSs.** The development of TLSs, from induction and initiation to maturation, is a highly orchestrated process. Under the influence of pro-inflammatory mediators, LTi cells interact with LTo cells. This interaction activates signaling pathways that trigger the production of downstream cytokines, which in turn regulate the maturation of TLSs.** (A) Induction of TLSs.** Various stimuli, including drugs, tobaccos, gut microbiota, and viruses, can induce the production of pro-inflammatory mediators, thereby promoting the recruitment of LTi cells and the development of TLSs. **(B) Initiation of TLSs.** LTi cells interact with LTo cells through various signaling axes, such as IL-7/IL-7R, IL-17/IL-17R, RANK/RANKL, and LTα1β2/LTβR, initiating cytokine expression and promoting the development of TLSs. These signaling pathways promote the secretion of VEGFA/C, adhesion molecules (*e.g.*, VCAM1, ICAM1, MADCAM1 *et al.*) and chemokines (*e.g.*, CXCL13, CXCL12, CCL21 *et al.*), aiding HEVs in the recruitment of lymphocytes into TLSs.** (C) Maturation of TLSs.** These cytokines intricately regulate cellular activities associated with TLS maturation at different status, including the recruitment of FDCs, compartmentalization of T/B cells, and the formation of GCs alongside B cell differentiation. This process is closely linked to the maturation of TLSs and the orchestration of immune responses. CAF: cancer-associated fibroblast; CCL21: CC motif chemokine ligand 21; CXCL13: CXC motif chemokine ligand 13; DC: dendritic cell; E-TLSs: early tertiary lymphoid structures; FDC: follicular dendritic cell; FRC: fibroblastic reticular cell; GC: germinal center; HEV: high endothelial venule; ICAM1: intercellular adhesion molecule 1; IL-17/IL-17R: interleukin 17/interleukin 17 receptor; IL-7/IL-7R: interleukin 7/interleukin 7 receptor; ILC: innate lymphoid cell; LTα1β2/LTβR: lymphotoxin α1β2/lymphotoxin beta receptor; LTi: lymphoid tissue-inducer; LTo: lymphoid tissue organizer; LV: Lymphatic vessel; MADCAM1: mucosal addressin cell adhesion molecule 1; PC: plasma cell; PFL-TLSs: primary follicle-like tertiary lymphoid structures; RANK/RANKL: receptor activator of nuclear factor κ B/receptor activator of nuclear factor κ B ligand; SFL-TLSs: secondary follicle-like tertiary lymphoid structures; T_h_ 17: T helper 17; T_fh_: T follicular helper; T_reg_: regulatory T cell; VCAM1: vascular cell adhesion molecule 1; VEGFA/C: vascular endothelial growth factors A/C; VSMC: vascular smooth muscle cell.

Similar to SLOs, the formation of TLSs originates from the homing of lymphoid tissue inducer (LTi) cells or their substitute cells to inflammatory sites 10. Extensive research has demonstrated that various persistent antigenic stimuli, including drugs, tobaccos, gut microbiota, and viruses, can induce the production of pro-inflammatory mediators, thereby promoting the recruitment of LTi cells and the development of lymphatic structures (Figure 3A) 34-37. Under the influence of pro-inflammatory mediators, LTi cells interact with lymphoid tissue organizer (LTo) cells. This interaction activates signaling pathways such as lymphotoxin α1β2/lymphotoxin beta receptor (LTα1β2/LTβR), interleukin-7/ interleukin-7 receptor (IL-7/IL-7R), IL-17/IL-17R, and RANK/RANKL, which trigger downstream cytokine production (Figure 3B) 38-40. Recently, one study found that in PDAC, IL-33 activates group 2 innate lymphoid cells (ILCs-2) expressing LT 41. These ILCs-2 interact with LTβR^+^ myeloid organizer cells, contributing to the production of downstream factors and TLS formation 41. Such cytokines intricately regulate cellular activity associated with TLS development at different status, involving stromal cell activation in conjunction with the LTi-LTo positive feedback loop, HEV expansion and compartmentalization of T/B cells, as well as GC formation with B cell differentiation 17. This process is closely linked to the maturation of TLSs and the orchestration of immune responses (Figure 3C). However, it is important to note that in clinical observations, not all TLSs achieve full or optimal development, exhibiting variability in maturation status, localization, and density. Further studies have revealed that endogenous and exogenous factors influence signaling pathways and cellular activities during the induction, initiation, and maturation of TLSs. Current research has identified the STING pathway, LTα1β2/LIGHT pathway and other regulatory targets; however, the mechanisms underlying TLS heterogeneity remain to be fully elucidated.

### Maturation of TLSs

TLSs have been found to exhibit two distinct maturation status: mature and immature (Figure 1A) 10, 11, 42. The critical distinction between these states mainly resides in the presence of organized GCs 10, 11, 42. Previous clinical trials and animal studies have demonstrated that beyond the mere presence of TLSs, differences in their maturation status significantly influence prognoses for tumor patients and their responses to anticancer treatments 10, 11, 42. Mature TLSs enhance immune responses through coordinated activation of T and B cells, correlating with better response to ICB (*e.g.*, anti-PD-1/CTLA-4 therapies) and neoadjuvant chemotherapy (NAC) 9, 43, 44. However, compared to mature TLS, immature TLS may inhibit effector cell function, potentially leading to poorer clinical outcomes in patients receiving the same treatments 9, 43, 44. The following sections provide a detailed exploration of the definition of TLS maturation, factors influencing this process, and its implications for tumor immunity and clinical practice.

### Definition of TLS maturation

The maturation of TLSs can be divided into several status characterized by the accumulation and development of FDCs and GCs 10, 11, 42. In 1998, Wagner *et al.* first observed lymph node-like structures and GC presence in synovial tissues of rheumatoid arthritis patients 45. Among the 9 studied patients, CD23⁺ FDCs participating in GCs reactions were identified in 4 cases, while absent in the remaining 5, indicating structural heterogeneity in TLS composition 45. Subsequent studies revealed varying maturation status of TLSs in chronic inflammatory conditions and cancers, which may influence prognostic prediction in tumor therapy 6-11, 14, 18, 46. In 2008, Dieu-Nosjean *et al.* first reported the explicit presence of TLSs in cancer contexts and noted that the infiltration of mature DCs within TLSs correlated with favorable patient outcomes 46. Between 2020 and 2021, several groundbreaking studies further clarified that mature TLSs could predict the efficacy of ICB in solid tumors independently of PD-L1 expression 6-9. Immature TLSs, also known as early tertiary lymphoid structures (E-TLSs), exhibit loosely organized aggregates of T cells and B cells with few DC infiltration 10, 11, 42, 47. They are closely linked to T cell exhaustion, inflammatory activity, and immune suppression within the tumor microenvironment (TME) 47-49. In contrast, mature TLSs represent highly organized lymphoid aggregates characterized by B cell follicles encircled by T cell zones 10, 11, 42, 47. These structures can be classified as primary follicle-like (PFL) or secondary follicle-like (SFL) subtypes containing GCs 11, 49. In mature TLS, PFL-TLS also has T follicular helper (T_fh_) cells and FDCs networks that allow T cell immune activation and low-affinity antibody production 10, 11, 42, 49. SFL-TLS are characterized by the presence of GCs with GC B cells and FDCs allowing the production of memory B cells and high-affinity antibody secreting plasma cells (PCs) 10, 11, 22, 42, 49. Sometimes, TLS maturation represents not discrete status but a continuous evolution spanning immune activation to functional exhaustion. Recent studies employing spatial transcriptomics and pseudotime trajectory analysis have identified three distinct differentiation patterns of TLSs in hepatocellular carcinoma (HCC): mature, conforming, and deviating 50. Mature TLSs possess fully developed GCs with high expression of key genes such as *CXCL13*, supporting the differentiation of B cells into antibody-secreting cells. Conforming TLSs, despite lacking classical GCs, demonstrate B cell differentiation trajectories highly aligned with those of mature TLSs. Mature and conforming TLSs display gradient expression continuity of genes like *CXCL13* and *AICDA.* However, deviating TLSs exhibit interrupted B cell differentiation and defective HEV development, creating an immunosuppressive microenvironment.

The detection and classification of TLS maturation in pathology and clinical practice constitute a significant topic in immunology. In pathological examinations, hematoxylin and eosin/hematoxylin-eosin-saffron (H&E/HES) staining combined with CD3, CD20, and CD23 immunohistochemistry (IHC) are utilized to determine the TLS status 9, 30, 33, 42, 51-53. Initial H&E/HES screening identifies visible lymphatic aggregates (≥50 cells) in viable tumor regions (excluding ulcerated/necrotic areas) 9, 53. In most cases, further maturation classification of TLSs relied on IHC and multiplex immunohistochemistry/multiplex immunofluorescence (mIHC/mIF) techniques to characterize cellular components 9, 53, 54. Concretely, for lymphoid aggregates without visible GCs, classification is determined by sequential CD20 and CD23 staining: CD20⁺ B cells with CD23⁺ FDCs indicate mature TLSs; CD20⁺ B cells without CD23⁺ FDCs suggest immature TLSs; CD20^-^ B cells indicate non-TLSs 9, 53, 54. Furthermore, lymphatic aggregates with visible GCs in initial H&E/HES staining can be directly classified as mature TLSs 53. Notably, when dealing with dense TLSs, single CD20 and CD23 IHC staining exhibits higher sensitivity and accuracy compared to CD20/CD23 mIHC staining 53. In clinical practice, the classic classification criteria for immature and mature TLSs summarized by Vanhersecke *et al.* represents a widely adopted strategy across multiple researches on TLSs 9, 31, 53, 54. Concretely, if a sample contains only immature TLSs without mature TLSs, it is classified as immature TLSs. If a sample contains mature TLSs or both mature TLSs and immature TLSs (even when the mature TLSs proportion is lower), it is defined as mature TLSs 9, 31, 53, 54. Additionally, mature TLSs undergo additional subclassification: cases containing PFL-TLSs with or without E-TLSs are classified as PFL-TLSs, while those demonstrating SFL-TLSs alone or coexisting PFL-TLSs and SFL-TLSs are uniformly categorized as SFL-TLSs 31, 33, 51, 55.

Statistical analyses reveal significant inter-individual and inter-tumoral heterogeneity in TLS maturation status 30, 32, 33. Among hepatocellular carcinoma (HCC) cases, 47% of tumors exhibited TLSs, in which E-TLSs, PFL-TLSs and SFL-TLSs account for 26%, 16%, and 5% of patients, respectively 33. Differently, in 90.8% esophageal squamous cell carcinoma (ESCC) patients, peri-tumor TLS was observed - including 74.7% E-TLSs, 54.1% PFL-TLSs, and 64.9% SFL-TLSs 30. A multicancer studies (covering 20 cancer types with 477 samples) revealed two independent differentiation pathways for antibody-secreting cells (ASCs): classical GC-dependent and alternative extrafollicular (EF) pathways, showing cancer-type specificity 32. In EF-dominant cancers (*e.g.*, HCC, head and neck squamous cell carcinoma (HNSCC)), TLS lacks GC structure; whereas in GC-dominant cancers (*e.g.*, colon adenocarcinoma (COAD), lung carcinoma (LC)), TLS typically presents mature structure 32.

### Factors contributing to TLS maturation

The current approaches have facilitated the regulation of B cell migration and TLS development *via* the administration of cytokines such as CXC motif chemokine ligand 13 (CXCL13), LTα1β2/ tumor necrosis factor alpha (TNF-α), and CXCL12 16, 17. Nevertheless, although these interventions can induce the formation of TLSs, there is a notable paucity of GC B cell infiltration. The TME contains potential barriers to TLS maturation, such as tumor-draining lymph nodes (TDLNs) and epigenetics.

TDLNs are LNs that receive and process lymph fluid originating from the tumor area 56. During the process of distant metastasis, tumors and their derivatives enter the TDLNs *via* afferent lymphatics, coinciding with vascular stroma remodeling and TME imbalance, which may influence the maturation of TLSs 56. A study highlighted that B_reg_s preferentially accumulate in TDLNs and promote tumor growth 57. However, unlike conventional B_reg_s, TDLN B cells exert immunosuppressive effects by inducing lymphangiogenesis in LNs rather than promoting IL-10 secretion or inducing regulatory T cell (T_reg_) differentiation 57. In addition, a study involving 218 patients with radically resected lung adenocarcinoma indicated that patients with lymph node metastasis typically exhibited immature TLSs and insignificant lymphocyte infiltration, prompting further investigation into the influence of TDLNs on TLS maturation 58. He *et al.* demonstrated that the immunosuppressive microenvironment of TDLNs blocked the maturation of TLSs and made TLSs lose its prognostic value 54. The immunosuppressive factors enriched in TDLNs obstruct the formation of memory B cells and interfere with interferon gamma (IFN-γ) signal transduction in NK cells 54. The interruption of IFN-γ signaling can synergize with immunosuppressive factors to affect the differentiation of memory B cells and the formation of GCs, thus limiting the development and maturation of TLSs 54.

Tumor cells adapt their metabolic pathways to meet the demands of rapid proliferation 59. These metabolic changes can influence the activity of epigenetic modifiers, leading to changes in gene expression patterns and contributing to an immunosuppressive environment 59. A pan-transcriptomic analysis involving 20 different cancer types indicated that increased glutamine metabolism in the TME promotes a bias in B cell differentiation towards an atypical memory (AtM) phenotype 32. AtM B cells are localized centrally within immature TLSs, displaying exhausted and bystander phenotypes, and serve as key contributors to the immunosuppressive microenvironment within these structures 22, 32. The study found that the glutamine-derived metabolite α-ketoglutarate facilitates the activation of AtM B cells by promoting the expression of transcription factors T-bet and BATF, as well as activating the mTORC1 signaling pathway 32. Furthermore, Bessode *et al.* demonstrated that the accumulation of the tryptophan catabolizing enzyme indoleamine 2,3-dioxygenase 1 (IDO1) within non-small cell lung cancer (NSCLC) induces an immunosuppressive state by converting tryptophan into various immunosuppressive metabolites, such as L-kynurenine 60. The study indicates that IDO1 is expressed in mature FDCs within TLSs and, through upregulating the transcription factors FOXP3 and Ki67, inhibits interactions between T_fh_ cells and B cells, thereby impairing plasmablast differentiation within mature TLSs 60. Similarly, spatial transcriptomic analyses of HCC reveal that malignant cells impede the TLS maturation by modulating chromatin accessibility and transcriptional activity of tryptophan metabolism-related genes 50. Malignant cells accumulate around immature TLSs, exhibiting increased promoter accessibility and upregulated expression of tryptophan metabolic enzymes, leading to the aberrant accumulation of tryptophan metabolites in the TME. These tryptophan metabolites inhibit the differentiation of B cells into GC B cells, thereby disrupting the normal maturation trajectory of TLSs.

### Role of TLS maturation in tumor immunity

The effects of TLSs in tumor immunity varies significantly depending on its maturation. Mature TLSs are typically associated with robust anti-tumor immune responses in cancer. Research indicates that B cells within mature TLSs may be a critical determinant of the efficacy of anti-tumor immunity 61. Within the GC of TLSs, naïve B cells differentiate into PCs that produce IgG and IgA, inducing macrophage/NK-cell-dependent tumor apoptosis 22, 62, 63. Furthermore, there is potential cross-talk between B cells and T cells that can modulate the efficiency of anti-tumor immunity 64-66. For example, CD86^+^ B cells clustered within TLS can present antigens to T cells, thereby inducing specific cellular immune responses 64. In addition to antigen presentation, B cells also exhibit regulatory effects on T cell phenotypes 65, 67, 68. A study on GC patients indicates that B cells within TLSs can promote the differentiation of naïve T cells into CD8^+^CD103^+^ resident memory T cells (T_rm_s) rather than FOXP3^+^CD8^+^ T_reg_s 67, 68. Importantly, the interaction between B cells and T cells may be bidirectional. T_fh_ cells secrete CXCL13, guiding B cell migration and promoting GC formation 66, 69. A recent study has revealed that in HNSCC, progenitor exhausted CD4^+^ T cells, with features resembling T_fh_ cells, support these responses, by activating B cells to produce PCs in the GCs, and interacting with DC-LAMP^+^ DCs to support CD8^+^ T cell activation 12. These findings indicate that mature TLS B cells and T cells work synergistically to enhance anti-tumor immune responses.

Immature TLS consist of loosely aggregated T cells, B cells, and stromal cells 10, 11, 42, potentially representing limited anti-tumor immune responses. In pancreatic ductal adenocarcinoma (PDAC), tumors with E-TLSs show significantly increased infiltration of CD3^+^ and CD8^+^ T cells compared to those without TLS 52. Although both immature and mature TLSs share comparable T cell infiltration, mature TLSs exhibit enriched CD4^+^ memory T cells and naïve B cells, alongside increased TMB and major histocompatibility complex (MHC) I neoantigens 52. Further research shows that E-TLSs tend to form an immunosuppressive microenvironment 48, 70. For example, in breast cancer (BC), B_reg_s and T_reg_s accumulate in E-TLSs, thereby maintaining this immunosuppressive state 70. In addition, pathological and gene expression profiles of 127 patients with early hepatopathy showed that the presence of E-TLSs was associated with increased expression of immunosuppression-related genes 48.

### The prognostic and predictive value of TLS maturation

Currently, a substantial number of clinical studies have found that the maturation of TLSs is associated with prognosis and treatment response in cancer patients. Mature TLSs exhibits positive prognostic and predictive value in various solid tumors, such as ESCC, clear cell renal cell carcinoma (ccRCC), urothelial carcinoma (UCC), and renal cell carcinoma (RCC) 22, 30, 43, 51. For example, in an analysis involving ESCC patients treated with the anti-PD-1 antibody nivolumab, mature TLSs was closely associated with better treatment responses and longer progression-free survival (PFS) 30. Similarly, in ccRCC, the presence of mature TLSs and GCs is significantly associated with better overall survival (OS) and PFS 51. Compared to E-TLSs, mature TLSs demonstrates higher infiltration of CD8^+^ T cells, CD20^+^ B cells, and DC-LAMP^+^ DCs 51. In contrast, E-TLSs often indicate poorer prognostic outcomes 30, 42, 51, 52, 70. In colorectal cancer (CRC), patients with a higher proportion of E-TLSs face increased risk of disease recurrence 42. E-TLSs exhibit lower MSI and fail to effectively induce immune activation 42. Similar findings have been reported in other solid tumors such as BC, ccRCC, ESCC, and PDAC 30, 51, 52, 70. Notably, the immunological role of E-TLSs is not entirely negative in a pan-cancer context 30, 52. For instance, one study on ESCC has indicated that the density of E-TLSs in the TME shows no clinical correlation with patient prognosis or responses to ICB therapy 30. Additionally, in PDAC, the presence of E-TLSs is correlated with prolonged PFS and OS 52. Tumors containing E-TLSs show higher levels of lymphocyte infiltration compared to those without any TLSs 52.

Furthermore, several key points about the clinical value of TLS maturation warrant emphasis. The maturation status of TLSs may be one of the most critical predictors of patient prognosis. TLS is commonly used as a parameter for predicting tumor patient survival 6, 71-73. However, research shows that TLS maturation has a deeper association with tumor prognosis compared to TLS appearance alone 74. When corticosteroids impair the formation of GCs, the predictive value of TLS presence is lost 74. Additionally, further research is needed to explore the prognostic differences between PFL-TLSs and SFL-TLSs. A study involving 138 patients with lung squamous cell carcinoma (LSCC) found that only the number or proportion of SFL-TLSs was significantly associated with improved survival, while the prognostic value of E-TLSs and PFL-TLSs remains unconfirmed 74. In intrahepatic cholangiocarcinoma (iCCA), mature TLSs shows a significant survival advantage over E-TLSs, but subdividing mature TLS into PFL-TLSs and SFL-TLSs revealed no additional prognostic differences 55.

### Localization of TLSs

TLSs can be observed within the TME, which includes the tumor core and stroma 10, 75. Based on their location within the TME, TLSs can be categorized into intra-tumoral, stromal, and peri-tumoral (*i.e.*, junctional) regions, with the majority of TLSs being located at the peri-tumoral areas (Figure 1B) 11, 33, 51, 76-79. Intra-tumoral TLSs are generally associated with enhanced responses to immunotherapies (*e.g.*, ICB, cancer vaccines, and CAR-T therapies), characterized by intact vascular networks and robust immune cell infiltration 67, 77, 80, 81. In contrast, peri-tumoral and stromal TLSs exhibit prognostic and predictive heterogeneity may due to vascular disruption and immunosuppressive microenvironments 33, 51, 76, 77, 82. Herein, TLS localization including its definition, regulatory factors, and its impact on tumor immunity and patient prognosis will be studied.

### Definition of TLS localization

TLSs localized within intra-tumoral, peri-tumoral, and stromal areas 11. Nevertheless, there exists no universally accepted criterion for defining the localization of TLSs. Strictly speaking, TLSs at the invasive margin are termed peri-tumoral TLSs, while those situated within the tumor stroma and distinctly separated from the tumor parenchyma are defined as stromal TLSs 11. Most studies do not distinguish between these two categories and commonly refer to both as peri-tumoral TLSs 30, 51, 76-79, 83. Some studies have more specifically described the presence of stromal TLSs within TME 24, 33, 82, 84, 85. Notably, the boundaries defining intra-tumoral versus peri-tumoral TLSs (including stromal TLSs) vary, with different studies setting distances from the invasive margin ranging from 0.5 millimeter to 10 millimeters 30, 51, 55, 78, 79, 83. The precise boundary between peri-tumoral and stromal TLSs is rarely quantified in clinical practice. For this reason, if not otherwise indicated, stromal TLSs are covered by the peri-tumoral TLSs described below.

Quantification of TLS localization relies on the intra-tumoral (T-score) and peri-tumoral (P-score) grading systems 26, 55, 77. The T-score employs a 0-3 grading scale based on the absolute count of TLSs within the tumor core 26, 55, 77. While the P-score classifies grades (0-3) according to the proportional area coverage of TLS in the tumor peripheral zone 26, 55, 77. However, no consensus exists for classifying cases simultaneously exhibiting intra-tumoral and peri-tumoral TLSs. Wu *et al.* proposed that if both intra-tumoral and peri-tumoral TLSs are observed, the patient should be considered intra-tumoral TLSs positive 76. On the other hand, Xu *et al.* have chosen to discuss this situation separately 51. Future studies need to standardize this debate. In clinical practice, combining T and P scores classifies patients into four immune subtypes (low-T/low-P, low-T/high-P, high-T/low-P, high-T/high-P), each featuring distinct TME and prognostic outcomes 26, 55, 79.

Existing evidence found that TLSs are more abundant in the peri-tumoral areas than in the core of tumors. Through pathological examination, in TLS-positive samples of various solid tumors (*e.g.* HCC, cSCC, ccRCC, iCCA, CRC), the proportion of intra-tumoral TLSs is approximately between 21% and 44%, while the proportion of peri-tumoral TLSs is roughly within the range of 56% to 79% 51, 76-79. Interestingly, the localization of TLSs also influences their morphological characteristics. Research indicates that in the same tumor tissue, different localizations of TLSs can lead to heterogeneity in their morphology 20, 77, 78. For example, Shang *et al.* showed that intra-tumoral TLSs in cholangiocarcinoma (CCA) were generally oval-shaped and well-developed, whereas peri-tumoral TLSs appeared squished, slender, or simply lymphatic aggregates 77. Similar results have been reported in several studies concerning CRC 20, 78. The specific mechanisms underlying these phenomena require further investigation.

### Factors contributing to TLS localization

Existing evidence strongly indicates a close association between TLS localization and the state of tumor vasculature 55, 76, 86-88. Under appropriate immune stimulation, TLSs tend to develop at the intersections of microvasculature within the tumor 87. As the TME undergoes remodeling, functional vascular networks are disrupted, leading to the migration of TLSs along the invasive front to peri-tumoral or stromal regions 88. A study involving 308 patients with pancreatic cancer (PC) has demonstrated that tumor tissues harboring intra-tumoral TLSs exhibit a higher number of CD31^+^ endothelial cells and exhibited elevated levels of vascular endothelial-cadherin expression 87. Conversely, in tissues characterized by peri-tumoral TLSs or the absence of TLSs, vascular stability and maturation tend to be diminished 87. Similarly, research across other malignancies, including iCCA, melanoma, and HNSCC, has confirmed that intra-tumoral TLSs possess a more intact vascular system compared to peri-tumoral TLSs 55, 76, 86. Collectively, these data support the notion that vascular normalization facilitates the intra-tumoral localization of TLSs.

Given the pivotal role of vascular normalization, exploring potential factors that modulate this process such as STING and LIGHT signaling 89, 90, may reveal potential directions for regulating TLS localization. STING and LIGHT signaling pathways not only regulates the transcription of adhesion molecules like PNAd and MADCAM1 on endothelial cells but also involves the release of chemokines such as CXCL10, CXCL13 91-94. These adhesion molecules and chemokines work together to mediate the homing of lymphocytes and maintain the normal function of the vascular system 91-94. For instance, a hydrogel platform for co-delivery of chitosan (a STING agonist) and CpG (a TLR9 agonist) to stimulate the development of vascular networks 89. The study found that the synergistic activation of STING and TLR9 signaling significantly promotes the migration of immune cells to tumor sites and accelerates the formation of intra-tumoral TLSs 89. In addition, the combination of anti-fibrotic drugs with LIGHT-coding plasmids represents a promising strategy for reshaping the vascular matrix and inducing intra-tumoral TLSs 90. Concretely, antifibrotic drugs reversed the abnormal activation of fibroblasts and reduced collagen deposition in vessels; the LIGHT encoding plasmid upregulated adhesion molecules involved in endothelial-lymphocyte interactions, promoting the infiltration of cytotoxic T lymphocytes (CTLs) 90. Notably, systemic STING and LIGHT application may pose risks of immune-related adverse events (irAEs) and immune cell off-target toxicity, highlighting the importance of precise control over signal expression 90, 95, 96.

### Role of TLS localization in tumor immunity

The localization of TLSs directly correlates with their immunological efficacy in TME. When TLSs are located within the tumor, cancer patients generally exhibit enhanced immune response 26, 51, 76, 87, which might be associated with an intact vascular network. The vascular structures related to TLSs form physical barriers that effectively limit the invasion and metastasis of tumor cells 26, 55, 76, 77, 83. For instance, in cSCC, intra-tumoral TLSs correlate with reduced subcutaneous fat penetration, decreased lymphatic vasculature, and reduced perineural invasion 76. Furthermore, the vascular system associated with TLSs plays a significant role in promoting lymphocyte infiltration 26, 55, 78, 87. Compared to peri-tumoral TLSs, the PDC tissues with intra-tumoral TLSs exhibit less vascular disruption, higher infiltration of T and B cells, as well as significantly higher expression of T helper 1 (T_h_ 1)- and T_h_ 17-related genes 87. It should be noted that the vascular system associated with TLSs non-selectively recruits immune cells, including T_reg_s and M2 macrophages 26, 55, 78. Additionally, intact vascular networks within intra-tumoral TLSs enhance immune cell recruitment, potentially contributing to TLS maturation processes 30, 33, 51, 55. A retrospective analysis of 395 ccRCC patients indicated that proximal TLSs are mainly composed of SFL structures, while distal TLSs have a higher proportion of early TLSs 51. Similar results have been observed in ESCC, iCCA, and HCC 30, 33, 55.

As the tumor invasion progresses, TLSs shift to the stromal regions farther from the tumor core and may be less affected by vascular immunity 33, 55, 83, 97. In this case, the infiltrated immune cells in TME exhibit considerable heterogeneity. For example, in BC, the invasive margins accumulate higher density of CD163^+^ M2 macrophages compared to the tumor core 83. These M2 macrophages contribute to abnormal angiogenesis and tumor metastasis by secreting factors such as IL-4, IL-10, and VEGF 98. In addition, a study covering 170 HCC patients noted that the denser peri-tumoral TLSs were linked to an increased infiltration of neutrophils 97. These neutrophils secrete mediators including α-defensins and transforming growth factor beta (TGF-β), which inhibit T cell activation and promote tumor cell proliferation 99, 100. Notably, in patients with iCCA, increased peri-tumoral TLS density positively correlates with elevated T_reg_ infiltration in intra-tumoral TLSs, implying immunological communication between distinct TLS niches 55. However, some studies found that the presence of peri-tumoral TLSs can also favor anti-tumor immunity 30, 101. In ESCC, mature peri-tumoral TLSs, particularly those characterized by GC B cells, are associated with a stronger anti-tumor immune response 30. Moreover, CD83^+^DC-LAMP^+^ DC clusters show a peri-tumoral preference in positioning across various solid tumors, such as melanoma, BC 101, 102. Mature DCs activate T cells through antigen presentation 101, 102. T cells gather around mature DCs in the peri-tumor area, forming clusters of DC-T cells that resemble SLOs, which are characteristic of sustained immune response 101, 102. Nevertheless, the exact mechanisms by which these critical immune cells are maintained during vascular invasion and TLS metastasis localization remain unclear.

### The prognostic and predictive value of TLS localization

The therapeutic implications of TLSs in cancer are heavily influenced by their localization. The available evidence found that intra-tumoral TLSs may have better prognostic and predictive significance 26, 33, 76, 103. In gastric carcinoma (GC), tumor resection specimens from responders exhibited a significantly higher number of intra-tumoral TLs compared to non-responders 67. These intra-tumoral TLSs are characterized by enriched infiltration of CD8^+^ exhausted T cells, which exhibit responsiveness to anti-PD-1 therapy and can unleash their anti-tumor potential 67. In NSCLC, the presence of intra-tumoral TLSs is closely associated with better DFS and OS 103. The intra-tumoral TLSs showed a higher proportion of switched memory B cells and a lower proportion of naïve B cells, supporting specific humoral immunity 103. Similarly, intra-tumoral TLS has also been shown to be a favorable prognostic and predictive predictor for other tumors, including CCA, CRC, HCC, and cSCC 33, 76, 77, 103. In contrast, the presence of peri-tumoral TLS is generally associated with a higher risk of cancer recurrence and negative treatment response 51, 76, 77, 82. For example, a study on CCA, a higher density of peri-tumoral TLSs was associated with a shorter 5-year OS in patients undergoing surgery or anti-PD-1 therapy 77. Peri-tumoral TLSs in H&E stained sections usually appear as squished, slender, or simply lymphatic aggregates lacking mature structure 77. Finkin *et al.* found that patients with a large number of hepatic stromal TLSs have a higher likelihood of late recurrence and mortality following HCC resection 82. Study showed that stromal TLSs within HCC act as niches providing cytokines such as IL-6, LTα, and LTβ, supporting the survival and growth of tumor progenitor cells 82. Notably, different prognostic and predictive value of peri-tumoral TLS have also been reported 30, 33. In a study covering 34 ESCC cases, the density and maturation status of peri-tumoral TLSs emerged as valuable parameters for predicting long-term survival and anti-PD-1 therapy response 30. However, another study involving 273 HCC patients reported no association between stromal TLSs and patient prognosis 33.

However, the clinical value of TLS localization may demonstrate heterogeneity across tumor types and disease progression stages. For instance, in HCC, the density of intra-tumoral TLSs is correlated with lower recurrence risk in early-stage patients, yet exhibits limited prognostic value in advanced stages 33. Furthermore, the immunological classification based on T/P combined scores lacks a unified guideline in pan-cancer contexts 26, 55, 79, 87. Several studies on PDAC and HCC have indicated that the combination of high T score and high P score is a key marker for optimal prognosis 79, 87. This contrasts with the traditional view that a high T score coupled with a low P score is advantageous 26, 55. Future studies should explore the practical significance of TLS localization across various cancers, to improve personalized prognosis in clinical practice. On the other hand, there was a significant association between the intra-tumoral localization of TLSs and its maturation status, both of which independently predicted positive clinical outcomes 30, 33, 51, 55. Although there is currently a lack of systematic studies to verify their synergistic effects, integrative analysis may optimize the accuracy of prognostic models.

### Density of TLSs

The presence and density of TLSs vary considerably across different cancer types and individual patients (Figure 1C) 10, 11, 18. High TLS density is closely associated with the enrichment of mature DCs, effector T/B cells, and the development of HEVs 51, 104-106. This correlation typically predicts better prognosis in cancer patients undergoing various treatments, including surgery, radiotherapy, chemotherapy, and immunotherapy 42, 73, 87. But negative reports exist regarding the correlation between TLS density and patient prognosis 55, 69, 107, likely attributable to compositional heterogeneity in TLSs and the absence of standardized quantification criteria. Therefore, elucidating how TLS density is defined, what factors regulate it, and how it shapes both TME and clinical outcomes remains crucial.

### Definition of TLS density

Currently, the definition of TLS density includes absolute TLS counting and the proportional area analysis of TLS. Absolute TLS counting involves characterizing the density of TLSs within a defined region by expressing the number of TLSs per square millimeter 30, 33, 78, 79. This method is favored for its robustness and interpretability in the pan-cancer context. Furthermore, some studies opt for specific cell types, such as DC-LAMP^+^ DCs, as indicators of TLS presence, particularly in NSCLC research 46, 85. Similarly, other studies have utilized B cell aggregates or HEVs as proxy markers for quantifying TLSs 108-110. Despite this, these cell composition-based counting methods have not been sufficiently validated across a broader range of cancer types. The proportional area analysis involves normalizing the total area covered by TLSs relative to the entire tumor region to assess the distribution density of TLSs 24, 26, 87. While this approach simplifies the evaluation of TLS density, it also compromises precision and reproducibility to a certain extent.

Multiple strategies provide feasible options for quantifying TLS density; however, determining the high and low density of TLSs remains a challenge. A widely applied strategy is the four-tier TLS scoring system 55, 77, 103. This scoring system defines four distinct grades corresponding to the absence, minimal presence, moderate presence, and extensive presence of TLSs 55, 77, 103. Nonetheless, this scoring system exhibits a degree of subjectivity, requiring the standardization of cutoff values for each grade and validation of their consistency across different tumor contexts. A more simplified alternative involves adopting a binary model to categorize TLS density as either high or low 30, 33, 72-74, 85. In this approach, the threshold defining high and low TLS density varies across studies. Some studies opt to use the median total TLS density as the baseline for stratification 30, 33, 72, 73. Others employ strategies such as the minimum p-value method or AUC-based ROC curve analysis to determine and validate the validity of the threshold 74, 85. These approaches not only help mitigate biases arising from inter-individual variability but also enhance the consistency and comparability of results across different studies 74, 85.

Studies have shown that TLSs can be detected in most types of solid tumors 10, 11, 18, but their distribution characteristics vary significantly across cancer types and populations. For example, the TLS positivity rate in BC ranges from 37% to 39%, while CRC and ESCC exhibit much higher positivity rates of 80%-90% 28-31. Distinct tumor types also demonstrate differences in TLS density. More aggressive cancers like UCC, ESCC show median TLS density of 0.16-0.36/mm², whereas low-infiltrative HCC maintains median density below 0.06/mm² 30, 33, 73. Furthermore, the heterogeneity of TLS density was more prominent in metastatic lesions. TLS density in lung metastases vary widely, with CRC and prostate cancer (PCa) metastases exhibiting high levels and leiomyosarcom and osteosarcoma metastases showing minimal presence 10, 111. Notably, TLSs remain undetectable in brain metastases of melanoma and BC 62, 112.

### Factors contributing to TLS density

Several widely employed cancer therapies, including chemotherapy, radiotherapy, ICB, tumor vaccine and oncolytic virus (OV) have been shown to trigger TLS accumulation within TME 6, 37, 44, 113, 114. Chemotherapy can promote the infiltration of immune cells into the tumor bed, induce immunogenic cell death, and exert beneficial effects on the accumulation and function of TLSs 115. Zhang *et al.* found that bladder cancer (BCa) patient treated with chemotherapy exhibited a higher abundance of CD20^+^ B cells, T_fh_ cells, and TLSs compared to the treatment-naïve patient 44. Similarly, Lu *et al.* reported that neoadjuvant chemotherapy in BC induces a subset of ICOS-L^+^ B cells expressing complement receptor CR2, which is associated with TLSs development and improved DFS and OS 116. It merits emphasis that, while corticosteroids are frequently co-administered with chemotherapy to alleviate adverse reactions, their prescription demands caution. Research indicated that corticosteroids may induce the reduction of TLSs density in TME, potentially compromising the beneficial clinical outcomes associated with these structures 43, 74.

In contrast, the impact of radiotherapy on immune cells and TLSs is more complex. Local radiotherapy can stimulate the adaptive immune response crucial for TLS functionality by increasing the expression of MHC I and co-stimulatory molecules 117, 118. However, some studies found that radiotherapy can transiently inhibit CD8^+^ T cells and enhance T_reg_ infiltration, creating an immunosuppressive microenvironment 119. Boivin *et al.* observed that hypofractionated radiotherapy initially led to a decrease in TLS density, which recovered within two weeks 113. This finding further illustrates the dynamic influence of radiotherapy on TLS formation, with specific mechanisms warranting further investigation.

By targeting pathways such as PD-1/PD-L1 and CTLA-4, ICB therapy reinvigorates T cell-mediated anti-tumor responses and bolsters immune memory, providing a supportive TME for the formation and accumulation of TLSs 2. Multiple studies confirmed that in a variety of solid tumors such as melanoma, UCC, and RCC, patients who responded to anti-PD-1 therapy showed denser tumor-infiltrating lymphocytes (TILs) and significant accumulation of TLSs 6, 43, 120. Of note, Helmink *et al.* specifically focused on the impact of ICB on B cell populations in TLSs 6. They revealed that TLSs-related B cells increased significantly in patients with high-risk melanoma and RCC who received ICB therapy. These B cells cooperate with other key immune components in TLSs to jointly optimize the immune efficacy of TLSs by altering T cell activation and function as well as through other mechanisms 6.

Tumor vaccines induce adaptive immune response through the use of tumor cells or their antigens, thereby inhibiting tumor growth, spread, and recurrence 121. Recent studies have shown that therapeutic vaccination can promote the generation of TLSs in tumors with low immunogenicity 114, 122. For instance, in patients with high-grade cervical intraepithelial neoplasia (CIN) treated with human papillomavirus oncoprotein vaccines, the regression of lesions correlates with the formation and clonal expansion of TLSs 114. Similarly, Lutz *et al.* used a combination of irradiated allogeneic granulocyte-macrophage colony-stimulating factor-secreting PDAC vaccine with cyclophosphamide, successfully inducing T cell infiltration and the development of TLSs, transforming “cold” tumors into “hot” ones 122.

OV is a type of natural or genetically modified virus that can selectively infect and kill tumor cells, causing less damage to normal cells 123. However, the role of OV in the TLS formation remains to be clarified. A recent study has pointed out that oncolytic herpes simplex virus-1 (oHSV) induces TLS formation in 4MOSC1 and MC38 subcutaneous tumor models, and increases B cell infiltration and TCF1^+^CD8^+^ T cell proliferation 37. Mechanistically, oHSV increases the expression of TLS-related chemokines and simultaneously upregulates CXCL10/CXC motif chemokine receptor 3 (CXCR3) to promote TLS formation. Furthermore, oHSV-mediated TLS formation revealed superior response and survival rate when combined with aPD-1 treatment. Another study revealed that in ICB refractory HNSCC, oncolytic adenovirus induces TLS characteristics and enhances anti-tumor immunity 124. The transcriptome analysis demonstrated that oncolytic adenovirus treatment induced TLS-associated gene signatures (*e.g., CXCR5, LTA, LTB*), increased B cell activation markers CD19 and immunoglobulin synthesis-related genes.

### Role of TLS density in tumor immunity

In various cancers, such as HNSCC, GC, BC, BCa, an increase in TLS density correlates with enhanced TILs activity 44, 72, 86, 109, 110. Compared with SLOs, TLSs, as non-encapsulated units, can more directly capture tumor antigens and pro-inflammatory mediators, thus accelerating local immune activation 11. Notably, multiple studies on solid tumors such as LSCC, CRC, and ESCC have demonstrated that patients with different TLS density exhibit heterogeneity in TLS maturation 30, 42, 74. Concretely, TLSs^low^ tumor predominantly featuring E-TLS structures, whereas TLSs^high^ tissues show more mature TLSs 30, 42. Mature TLSs may create a supportive immune microenvironment that promotes its accumulation, requiring experimental confirmation. While TLSs demonstrate potent anti-tumor immune potential, studies also report that TLSs can become immune-tolerant niches for malignant cells 70, 107, 125, 126. For instance, in a CCL21-engineered melanoma model, FOXP3^+^ T_reg_s and myeloid-derived suppressor cells (MDSCs) were recruited to TLSs, promoting tumor growth 126. Furthermore, other cell types such as M2 macrophages, T_h_ 2 cells, and B_reg_s have been identified as contributors to the immunosuppressive microenvironment within TLSs 70, 107, 125. Notably, several studies indicate that this immunosuppressive state within TLSs extends beyond the suppression of anti-tumor immunity and may directly support tumor growth and metastasis through the secretion of cytokines 28, 82.

Despite the presence of immunosuppressive cells, a higher density of TLSs is generally associated with enhanced anti-tumor immunity across most cancer types 51, 79, 85-87. This may be attributed to the mature CD83^+^DC-LAMP^+^ DCs dominated TIL crosstalk. CD83^+^DC-LAMP^+^ DCs are ubiquitously present within TLSs, including those that are immature or located peripherally to the tumor core 30, 46, 48, 51, 74, 127. These cells possess an immunomodulatory potential that does not vary with the heterogeneity of the TLSs. CD83^+^DC-LAMP^+^ DCs are localized within the T cell compartments of TLSs, where they activate the differentiation of naïve T cells *via* MHC-mediated antigen presentation 22. In addition to T cell activation, several studies found a positive correlation between CD83^+^DC-LAMP^+^ DCs and GC B cells, NK cells, and HEVs 46, 68, 106, 111, 128, 129. For instance, in murine models, CD11c^+^ DCs (specific mouse marker) can induce the development of the vascular system and promote TLS assembly by secreting LTβ or activating the STING signaling pathway 104, 105. Furthermore, in BC, mature DCs and HEVs can co-develop despite the presence of T_reg_s 106, further substantiating the effectiveness of DC-mediated immune coordination in combating immune tolerance.

### The prognostic and predictive value of TLS density

In various human cancers, higher TLS density is correlated with prolonged patient survival and improved response to ICB 6, 42, 74, 85, 87. This finding has been extensively reviewed and summarized elsewhere 10, 11, 14, 18. Despite this overall positive correlation, negative relationships between TLS density and patient outcomes have also been reported 55, 69, 107. For instance, Ding *et al.* identified T_reg_ -skewed TLSs in iCCA, where their density strongly correlated with poorer 5-year OS 55. T_reg_s suppress anti-tumor immune responses by disrupting co-stimulatory signals between antigen-presenting cells (APCs) and effector T cells, and secreting immunosuppressive molecules 130. One possible reason for the difference in the resulting data is a lack of consensus on what constitutes TLSs and how laboratory quantify them 20. So far, the definition of TLSs has varied in each study 46, 76, 101, 102, 110. Several studies have identified TLSs as DC-LAMP^+^ mature DC aggregates 46, 101, 102, while others define them as CD20^+^ B cell clusters or other immune aggregates 9, 76, 110. TLSs are not functionally homogeneous immune aggregations, their cellular composition directly determines anti-tumor immune efficacy and clinical outcomes 6, 23, 55, 107, 125, 131. For instance, in melanoma, elevated B cell infiltration and TLS density were observed in responsive tumors treated with anti-PD-1 alone or combined with anti-CTLA-4 6. Conversely, in a retrospective analysis of soft-tissue sarcomas (STS) samples from a phase II trial of the anti-PD-1 antibody pembrolizumab, high TLS-associated T_reg_ infiltration was correlated with reduced objective response rates and poorer survival outcomes 131. These findings demonstrate that the cellular composition of TLSs differentially modulates anti-tumor immune responses across cancer types (Table 1).

Therefore, to establish the clinical value of TLS density in practice, it is essential to consider the heterogeneity and balance of cellular components within TLSs. Exploring the clinical significance of TLS classification based on their cellular components may represent a promising strategy 63, 107, 132. A study on ovarian cancer (OC) categorized TLSs into four types based on size, cellular composition, and GC organization 63. While investigators examined correlations between these TLS types and TIL (specifically PC) density, clinical outcome associations remained unexplored 63. In BCa, TLSs are classified into C1-C5 subtypes based on the expression profiles of 39 TLS gene signatures 132. These subtypes exhibit marked heterogeneity in TME and prognostic outcomes 132. The C2 subtype, characterized by robust infiltration of B cells, CD8^+^ T cells, and T_fh_s, is associated with optimal survival outcomes 132. In contrast, the C4 subtype (with elevated CCL20 expression) and C3/C5 subtypes (dominated by T_reg_s or stromal cells) correlate with poorer prognosis, indicating immune escape or a cancer-promoting microenvironment 132. Another research concerning CRC classified TLSs into five subtypes through quantitative analysis of six immune cell populations including T_h_ cells, GC B cells and FDCs 107. The GC-TLS, B cell-rich, and FDC-rich types have similar structural characteristics and favorable prognostic significance to mature TLS based on CD21/ CD23 classification 107. Notably, T_h_ 2-enriched TLSs exhibited skewed distribution in recurrent patients, indicating potential immunosuppressive imbalance in TME 107. These advances indicate that establishing a unified standard across cancer types is important—requiring integration of TLS density and quantitative analysis of key functional cells, thereby achieving progress from precise prognostics to mechanistic intervention.

### Evaluation of TLS heterogeneity

The efficacy heterogeneity of ICB has driven the exploration of more precise biomarkers. In recent years, TLSs, as critical hubs of anti-tumor immune responses within TME, have gradually garnered clinical attention 10, 11, 18. However, the biological functions of TLSs and their synergistic or complementary relationships with other biomarkers have yet to be systematically elucidated. Moreover, the inherent heterogeneity of TLSs poses technical challenges to traditional assessment methods, necessitating the development of advanced analytical tools.

### Joint evaluation of TLSs and other biomarkers

Evaluating the relationship between TLS metrics and other immunotherapy biomarkers (*e.g.*, TMB, neutrophil-to-lymphocyte ratio (NLR), PD-L1 expression) can uncover whether TLSs possess synergistic or complementary value in predicting immunotherapy outcomes 71, 133, 134. This approach may enhance response prediction compared to any single biomarker alone.

TMB alters protein structures on a genetic level, generating neoantigenic epitopes that may potentially promote immune activation and the development of TLSs 135. TMB, defined as the total number of base substitution mutations and indels per megabase across the tumor genome, has shown broad correlations with the efficacy of various cancer immunotherapies 136. Posch *et al.* revealed a positive association between BRAF mutations and the density and maturation of TLSs in 109 patients with stage II/III nmCRC 42. This sparked deeper investigation into potential links between TMB and TLSs. Yet, several studies noted the absence of direct statistical correlation between TLS density and TMB in melanoma, BCa, and muscle-invasive bladder cancer (MIBC), both factors independently associated with patient survival 7, 132, 133. Notably, Pagliarulo *et al.* confirmed the significant predictive value of combining TMB and TLSs assessment for patient prognosis 133. The combination of high TLS density and elevated TMB was associated with the most favorable OS, indicating a potential synergistic biomarker effect 133. Given the uncertainty of the association between TLSs and TMB in different cancer contexts, their relationship warrants further investigation.

Elevated levels of neutrophils can suppress lymphocyte and NK cell activation, potentially inhibiting the anti-tumor response mediated by TLSs 137. NLR, established as a systemic inflammatory marker, has been confirmed as an independent prognostic factor for various malignant tumors 136. Fukuhara *et al.* demonstrated a correlation between low blood NLR and high TLS expression in 147 NSCLC patients 71. In addition, this potential relationship between NLR and TLS density has also been corroborated in GC patients 109. However, some studies have indicated that there is no association between NLR and TLSs in uLMS and UCC 72, 138. Despite ongoing debates regarding TLSs and NLR as independent prognostic factors, combined assessment has identified cancer patients with the most favorable prognosis. Multiple studies shown that Patients with high TLSs and low NLR have consistently shown a survival advantage over those assessed by single indicators 72, 109, 138.

Pre-existing expression of immune checkpoint molecules (*e.g.*, PD-L1) within the TME establishes a biological foundation for ICB efficacy 3. Extensive research indicates that TLSs independently predict ICB responsiveness, irrespective of checkpoint molecule expression levels 11. This raises critical questions about potential synergistic interactions between TLSs and immune checkpoints. Deng *et al.* first proposed combining TLSs with PD-L1 status as a composite biomarker framework for immunotherapy in primary cardiac angiosarcoma (PCA) 134. Their analysis demonstrated that even immature TLSs, when combined with PD-L1 positivity, guided anti-PD-1 therapy and correlated with transient metastatic LN regression 134. However, research on TLS-immune checkpoint synergy remains preliminary, requiring further validation.

### Cutting-edge technologies for TLS evaluation

**Figure 4:**
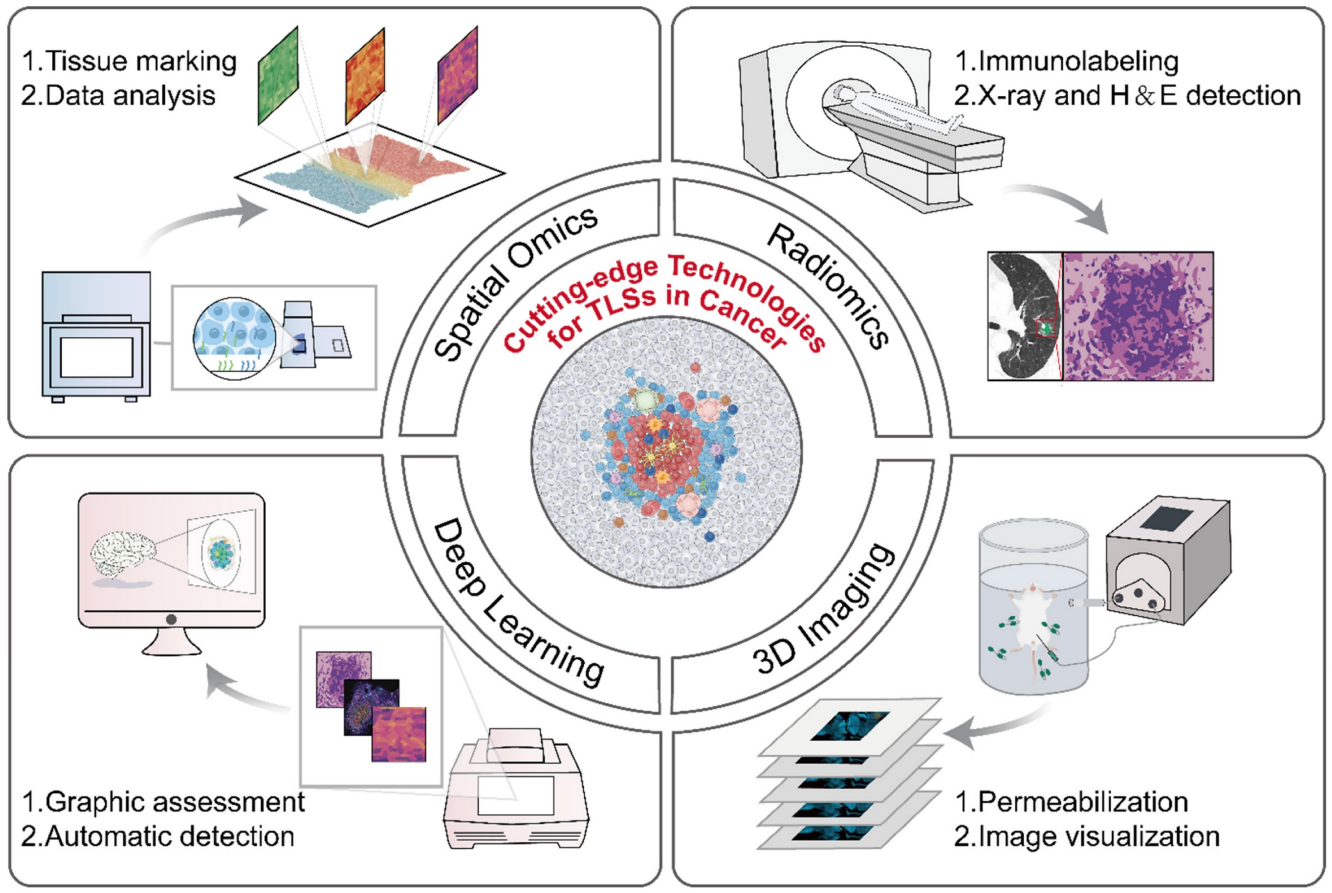
** Cutting-edge technologies for TLS evaluation**. The development of advanced technologies, such as radiomics, deep learning models, and three-dimensional (3D) imaging, etc., has overcome the limitations of traditional methods and propelled the advancement of the TLS research field. Spatial omics: High-throughput sequencing, microscopic imaging localization, quantification of tissue molecular expression. Radiomics: Extract features from medical images, screen for disease-related indicators. Deep learning models: Preprocess datasets, design neural network architectures, and train models. 3D imaging technologies: Perform sample permeabilization, capture images, and reconstruct 3D models via software algorithms. 3D: three-dimensional.

Currently, multi-omics technologies including histopathology, genomics, and transcriptomics offer a range of options for the detection and quantification of TLSs (Table 2). Despite their practical value, these conventional methods still possess several limitations, such as insufficient depth of information provided, the necessity for destructive testing, and complex operational procedures, all of which highlight the pressing demand for innovative technologies to tackle TLS heterogeneity effectively. The development of advanced technologies, such as radiomics, deep learning models, and three-dimensional (3D) imaging, etc., has overcome the limitations of traditional methods and propelled the advancement of the TLS research field (Figure 4) 7, 139-141.

Spatial omics technologies, including spatial transcriptomics, proteomics, etc., have become key tools for elucidating the relationship between TLSs and tumor immunity 7, 142. These technologies allow researchers to measure and map the expression of genes and proteins at specific localizations (*i.e.*, “spatially”) within tissue sections, enhancing our understanding of cell interactions and their impact on treatment responses 143. For instance, Cabrita *et al.* applied spatial proteomics to quantitatively analyze immune-related proteins in the TME 7. Their analysis revealed that T cells in TLS-deficient regions exhibited dysfunctional phenotypes, whereas TLS-associated T cells showed increased CD4^+^ proportions and elevated BCL2 (an anti-apoptotic protein) levels, demonstrating the critical role of TLSs in maintaining functional T cell states 7. In addition, a study using spatial multi-omics technologies unveiled the critical role of TLSs in the immune therapy response of HNSCC 142. Spatial proteomic analysis revealed significant upregulation of T lymphocyte markers (CD3, CD8), as well as PD-L1, in patients who responded to ICB, indicating a close association between the “hot tumor” phenotype and immune response 142. Further spatial transcriptomic analysis demonstrated that genes related to immune modulation, recruitment of various immune cells, and effective IFN response were markedly elevated in TLSs compared to normal GCs, indicating that TLSs harbor a distinct microenvironment for immune activation 142. Despite representing a significant advance in characterizing TLSs, spatial omics still faces challenges such as high costs, low throughput, and limited spatial resolution, necessitating technological innovation and optimization 143.

Compared to invasive detection methods, radiomics techniques, including computed tomography (CT), positron emission tomography (PET), single-photon emission computed tomography (SPECT), and magnetic resonance imaging (MRI) emerge as a promising non-invasive approach for the detection and assessment of TLSs 16, 139, 144, 145. This method utilizes computer technologies to extract and quantify high-level imaging features of tumors at high throughput and integrates this data with other clinical information to identify TLSs and their associations with tumor prognosis 146. For example, in a recent study, CT scans revealed multiple small solid components within partial solid nodules in lung adenocarcinoma (LUAD), findings that correlated with the presence of TLSs in histopathological examination 139. Using 99mTc-labeled albumin nanocolloid (99mTc-Nanocoll) as a tracer, Dorraji *et al.* successfully achieved non-invasive localization of TLSs in PC by SPECT 144. Histopathological analysis further confirmed that these regions exhibited characteristic pathological features, including T/B cell compartmentalization and macrophage infiltration 144. However, conventional imaging modalities (*e.g.*, CT, SPECT) remain limited by radiation exposure risks and insufficient soft-tissue resolution, constraining their clinical applicability 145. In contrast, MRI emerges as a superior alternative. For instance, a study on HCC developed an MRI-based predictive model by integrating intra- and peri-tumoral radiomic features 145. This model not only non-invasively assesses peri-tumoral TLS density distribution but also stratifies patients' survival outcomes and immunotherapy response profiles, offering a reliable objective basis for clinical decision-making 145.

Although these technologies provide non-invasive and real-time alternatives for the detection of TLSs, they still face challenges in terms of accuracy and specificity. In recent years, materials for single-cell imaging have been developed to improve detection accuracy 147, 148; however, whether imaging at the single-cell level can adequately characterize the presence of TLSs remains to be further investigated.

Deep learning models can automate the analysis and quantification of complex images and data related to TLSs, and demonstrate the potential to predict patient outcomes 140, 149-152. Recently, several deep learning models have been developed towards automated segmentation and quantification of TLSs from H&E images in various tumor types 140, 149, 150. Wang *et al.* devised an automated computational workflow to quantify the density of TLSs in routinely H&E-stained whole-slide images (WSIs) of LUAD tissue 140. Additionally, a Cox proportional hazard regression model, incorporating clinicopathological variables and the TLS density, was established to assess its prognostic ability 140. Similarly, deep learning models based on H&E images can be utilized to detect other parameters of TLSs, such as cellular composition and maturation, and these models have been extensively validated in different cancer settings 149, 150. While deep learning models based on H&E images currently dominate, alternative data-driven approaches also exhibit distinct advantages 151, 152. Unlike these studies that depended solely on pathologists' manual annotations of TLSs without mIHC guidance, Chen *et al.* leveraged mIHC markers—DAPI, CD3, and CD20—to identify TLSs, thus reducing the influence of subjective human judgment 151. In addition, Li *et al.* developed a machine learning model that used spatial transcriptomic data to identify markers of TLSs and effectively predict TLS localization, holding the promising potential to impact cancer treatment strategies 152. Particularly, the identified markers emphasize the significance of immunoglobulin genes in TLS detection, adding a novel perspective to existing knowledge 152. Deep learning models offer powerful tools for the efficient and automated analysis of TLSs, yet they face challenges in accurately distinguishing between LNs and TLSs, highlighting the need for future research to further improve model accuracy and reliability 153.

3D imaging technology offers a novel perspective for analyzing the spatiotemporal heterogeneity of TLSs 141, 154, 155. In CRC, whole-section 3D reconstruction has unveiled TLS networking features that conventional 2D pathology struggles to capture 155. A single TLS network can span multiple tissue layers and extend across millimeters 155. These 3D networks exhibit the gradient distribution of cellular structure (*e.g.* CD68⁺CD163⁺ macrophages, and B/T cells) and molecular markers (*e.g.* PD-L1, LAG-3, TIM-3), indicating that TLSs may play a dynamic coordinating role in anti-tumor immune response 155. A study on Crohn disease utilizing 3D imaging revealed significant anatomical associations between TLSs and mesenteric lymphatics 154. These B cell-enriched ectopic lymphoid structures are distributed along lymphatic pathways, especially in the fatty infiltrated mesenteric area, where TLSs embed directly into the lymphatic wall and trigger structural remodeling 154. Notably, IL-33R⁺ ILCs detected within some TLSs imply their potential involvement in lymphatic wall remodeling through cellular interactions 154. In addition, wildDISCO technology enables whole-body horizontal 3D imaging of mice through innovative whole-body transparency and multi-target immunolabeling strategies 141. Using dual labeling of CD23 and CD3 antibodies, this technique reveals the fine distribution of TLSs in primary tumors and lung and intestinal metastases in BC metastasis models 141. Subcellular-level imaging further demonstrates that even relatively large TLSs exhibit significantly smaller volumes compared to those in metastatic lesions 141.

## Conclusions and prospects

TLSs exhibit marked heterogeneity in maturation, localization and density across tumor types and individuals, profoundly impacting anti-tumor immune effects and clinical value (Table 3) 10, 11. Current evidence indicates that more mature, intra-tumoral, and higher-density TLSs serve as predictive biomarkers for enhanced response to ICB therapy and prolonged survival benefits 6, 18, 26, 30, 33, 132. Some studies have indicated that mature TLSs can also be localized in the peri-tumoral area, predicting a stronger anti-tumor immune response 30, 78, 79. Conversely, immature TLSs predominantly localized at the tumor periphery, low-density TLSs, or complete TLS absence are strongly associated with an immunosuppressive microenvironment, tumor progression, and unfavorable prognosis 6, 30, 33, 42, 55, 77. Building on these clinical observations, current studies have identified TDLNs, the STING pathway, and tumor vaccines, along with other potential targets, as key regulators of TLS heterogeneity 54, 89, 114. Significantly, the integrative analysis of TLS with other biomarkers such as TMB, NLR, and PD-L1 expression represents a novel prognostic prediction strategy 71, 133, 134. Equally important, emerging computational-driven technologies like spatial omics and radiomics enable efficient and comprehensive dissection of TLS-immune microenvironment interactions 7, 139-141. While the understanding of TLS heterogeneity has advanced considerably, translating these insights into clinical applications presents several challenges.

### Optimizing evaluation strategies for TLS heterogeneity

To effectively evaluate TLS heterogeneity, unified standards and comprehensive strategies must be established. Currently, there is a lack of standardized quantification criteria for TLSs across different patients and cancer types. Future studies should aim to define standardized criteria for spatial localization (e.g., distinguishing peritumoral from stromal regions) and establish uniform thresholds for classifying TLS density as high or low. For ambiguously defined spatial localization, an alternative strategy is to quantify TLS localization. This approach can be specifically manifested through calculating the precise distances from each target TLS margin to the tumor invasive margin or the interface between the tumor core and the tumor stroma. Through rigorous statistical analysis correlating continuous localization metrics with clinically meaningful endpoints (*e.g.*, OS, PFS, RFS, and response rates to ICB), clinically relevant minimal thresholds or critical points can be identified. Furthermore, the distance-clinical association curves can serve as references to refine the boundary values of conventional localization dichotomization or trichotomization strategies. Moreover, the quantitative relationships between TLS maturation, density, characteristic molecular expression levels and their clinical significance also remain unclear. Therefore, it is necessary to standardize the quantification of TLSs and define statistically significant minimum thresholds. Furthermore, multi-parameter integrated evaluation may serve as an effective strategy to enhance prognostic prediction accuracy. For instance, in CRC, the proportion of SFL-TLSs demonstrates a positive correlation with overall TLS density 42. A composite immune score incorporating both total TLS density and mature TLS density effectively stratifies patients with minimal recurrence risk 42. Other favorable TLS features also tend to cluster, such as intra-tumoral TLSs with higher maturation status, warranting further exploration of their combined clinical utility 30, 33, 51, 55. Moreover, integrating TLSs with other biomarkers such as TMB, NLR, and PD-L1 expression emerges as a promising prognostic prediction approach 42, 71, 134. However, the statistical associations between TLSs and other biomarkers vary across cancer types, necessitating further research to define clinical applicability.

### Advancing assessment technologies for TLS heterogeneity

Developing techniques suitable for assessing TLS heterogeneity represents a significant challenge. Recently, advanced technologies such as spatial omics, radiomics, deep learning models, and 3D imaging have emerged, overcoming the limitations of traditional methods and providing new options for the detection and evaluation of TLSs 6, 139-141. However, these technologies still face issues such as high costs, low throughput, and insufficient precision, which limit their widespread application. In the future, developing new evaluation technologies or integrating existing ones could be key to addressing these challenges. For example, the integration of radiomics and deep learning models has been used to characterize the microenvironment of GC and predict treatment responses 156. This approach maintains the advantages of non-invasiveness and high throughput, overcoming the limitations of single technologies.

### Developing materials for precisely regulating TLS heterogeneity

Given the role of TLSs in anti-tumor immunity, developing biomaterials capable of modulating TLS heterogeneity to enhance anti-tumor immune response is crucial. Various materials, such as cytokine-loaded nanoparticles, oncolytic virus vaccines, and bioscaffolds, have been developed to induce TLS formation, demonstrating initial feasibility 157, 158. However, current TLS induction methods are not yet mature, focusing primarily on the existence and density of TLSs rather than their precise maturation and localization. Moreover, some studies found that induced TLSs exhibit atypical states, requiring additional regulatory support 37, 158, 159. Future research should focus on identifying and regulating key factors influencing TLS heterogeneity to achieve precise management of TLSs, thereby enhancing tumor immune response and improving patient outcomes.

## Figures and Tables

**Table 1:** Immune cells in TLSs

Cell types	Cell subsets	Markers	Functions	Cancer types	Cases	Prognostic value	Predictive value to ICB	Ref.
Lymphoid cells	Naïve T cells	CD3^+^CD27^+^CD28^+^CD45RA^+^CD45RO^-^CD62L^+^CCR7^+^	Develop into functional T cells	Melanoma, LC	Mice	NA	NA	160
	T_h_ 1 cells	CD3^+^CD4^+^ Tbet^+^	Promote CTL differentiation	MIBC	153 patients	Association with longer OS	Positive	44
				GC	82 patients	Association with longer RFS	NA	161
				PDC	534 patients	Association with longer DFS, OS	NA	87
	T_h_ 2 cells	CD3^+^CD4^+^BCL6^+^GATA3^+^	Suppress T_h_ 1 and T_h_ 17 cell differentiation	CRC	67 patients	Association with shorter RFS	NA	107
	T_h_ 17 cells	CD3^+^CD4^+^RORγt^+^STAT3^+^	Promote TLS formation	ESCC	650 patients	Association with longer DFS, OS	NA	162
				PDC	534 patients	Association with longer DFS, OS	NA	87
	T_fh_ cells	CD3^+^CD4^+^ICOS^+^CXCR5^+^PD-1^++^	Activate GCs development	BC	70 patients	Association with longer DFS	NA	21
				MIBC	153 patients	Association with longer OS	Positive	44
				CRCLM	603 patients	Association with longer RFS, OS	NA	26
				iCCA	962 patients	Association with longer 5-year OS	NA	55
		CD3^+^CD4^+^TCF1^+^​​CXCL13^+^PD-1^++^		HNSCC	14 patients	NA	Positive	12
	T_fr_ cells	CD3^+^CD4^+^ICOS^+^CXCR5^+^PD-1^++^FOXP3^+^	Decrease GC B cells and CD8^+^ T cells infiltration	BC	179 patients	Association with shorter DFS, RFS	NA	69
	T_reg_s	CD3^+^CD4^+^FOXP3^++^ CD127^-^	Block T cells reactivation; reduce the TLS abundance	iCCA	962 patients	Association with shorter 5-year OS	NA	55
				GC	82 patients	Association with shorter RFS	NA	161
				STS	30 patients	Association with shorter RFS, OS	Negative	131
	T cells	CD3^+^CD8^+^CD28^+^CD39^+^CD45RA^+^CD45RO^-^	Secrete perforin and/or granzyme	ESCC	31 patients	NA	Positive	163
				UCC	45 patients	NA	Positive	73
	Memory T cells	CD3^+^CD45RA^-^CD45RO^+^CD62L^+^CCR7^+^	Maintain immune memory	NSCLC	458 patients	NA	NA	85
	T_rm_s	CD3^+^CD69^+^CD103^+^CD62L^-^CCR7^-^	Maintain immune memory; secrete CXCL13	GC	53 patients	Association with longer DFS, OS	Positive	67
				LUAD	49 patients	Association with longer DFS	NA	164
	Naïve B cells	CD20^+^IgM^+^IgD^+^CD27^-^CD38^-^	Develop into functional B cells	Melanoma	46 patients	NA	Negative	6
	GC B cells	CD20^+^CD27^-^CD38^+^Ki67^+^AID^+^BCL6^+^	Induce clonal expansion and somatic hypermutation; recruit T cells, DCs, macrophages, and NK cells	NSCLC	196 patients	Association with longer OS	NA	23
				HGSOC	570 patients	Association with longer OS	NA	63
				Melanoma	46 patients	NA	Positive	6
	Memory B cells	CD20^low^CD27^+^CD38^+/-^	Maintain immune memory	PDAC	39 patients	Association with longer OS	NA	52
				Melanoma	46 patients	NA	Positive	6
	B_reg_s	CD20^+^CD19^+^CD25^+^/IL-10^+^	Induce T_reg_s and TAMs activation	BC	489 patients	Association with shorter MFS	NA	70
	PCs	CD20^+^IgD^-^CD38^++^CD138^+^	Secrete anti-tumor abs	HGSOC	570 patients	Association with longer OS	NA	63
				RCC	59 patients	Association with longer PFS, OS	Positive	22
				STS	30 patients	Association with longer RFS, OS	Positive	131
	NK cells	CD56^+^NKp46^+^	Execute ADCC	HGSOC	167 patients	NA	NA	129
	ILCs-2	CD45⁺CD127⁺CRTH2⁺ KLRG1⁺	Induce TLS initiation	PDAC	328 patients	Association with longer OS	NA	41
	ILCs-3	NCR^+^	Induce TLS initiation	NSCLC	57 patients	NA	NA	165
Myeloid cells	DCs	CD80^+^CD83^+^DC-LAMP^+^	Promote T_h_ 1 cells, CTLs, and NK cells infiltration	ccRCC	186 patients	Association with longer DFS	NA	127
				Melanoma	82 patients	Association with longer OS	NA	102
				NSCLC	74 patients	Association with longer DSS, DFS, and OS	NA	46
		CD11c^+^		STS	30 patients	Association with longer RFS, OS	NA	131
	FDCs	CD21^+^ CD23^+/-^ MHC II^-^	Support follicular B cells and GCs in TLSs	HGSOC	570 patients	NA	NA	63
				NSCLC	196 patients	NA	NA	23
	Neutrophils	MPO^+^COX2^+^	Mediate inflammation; induce tumor angiogenesis	PC	17 patients	NA	NA	25
	Macrophages	CD64^+^CD68^+^ CD163^+^CD169^+^	Downregulate the GC reaction; recruit regulator cells	CRC	67 patients	Association with shorter RFS	NA	107
Other cells	FRCs	PDPN^+^VCAM1^+^	Direct PCs dissemination; provide structural support	RCC	59 patients	NA	NA	22
	CAFs	PDPN^+^FAP^-^ CCL19^+^	Orchestrate TLS formation	Melanoma	Mice	NA	NA	120
				CRC	Mice	Association with longer OS	NA	31
		hMENAΔv6^+^	Inhibit TLS formation	NSCLC	2006 patients	Association with shorter OS	Negative	103
	HEVs	PECAM1^+^PNAd^+^MADCAM1^+^	Recruit lymphocytes	TNBC	108 patients	Association with pCR	NA	110
	LECs	PECAM1^+^PDPN^++^LYVE1^++^VEGFR3^+^	Recruit lymphocytes	Melanoma, LC	Mice	NA	NA	160

ADCC: antibody-dependent cellular cytotoxicity; B_reg_s: regulatory B cells; BC: breast carcinoma; BCSS: breast cancer specific survival; CAFs: cancer-associated fibroblasts; ccRCC: clear cell renal cell carcinoma; CR: complete response; CRC: colorectal carcinoma; CRCLM: colorectal cancer liver metastases; CXCL13: CXC motif chemokine ligand 13; DCs: dendritic cells; DC-LAMP: dendritic cell-lysosomal-associated membrane protein; DFS: disease-free survival; DSS: disease-specific survival; ESCC: esophageal squamous cell carcinoma; FDCs: follicular dendritic cells; FRCs: fibroblastic reticular cells; GC: gastric carcinoma; HEVs: high endothelial venules; HGSOC: high-grade serous ovarian cancer; HNSCC: head and neck squamous cell carcinoma; ICB: immune checkpoint blockade; iCCA: intrahepatic cholangiocarcinoma; ILCs: innate lymphoid cells; LC: lung carcinoma; LECs: lymphatic endothelial cells; LUAD: lung adenocarcinoma; MFS: metastasis-free survival; MHC: major histocompatibility complex; MIBC: muscle invasive bladder carcinoma; NAC: neoadjuvant chemotherapy; NK cells: natural killer cells; NSCLC: non-small cell lung carcinoma; OS: overall survival; PC: pancreatic carcinoma; pCR: pathologic complete response; PCs: plasma cells; PDAC: pancreatic ductal adenocarcinoma; PDC: pancreatic ductal carcinoma; PR: partial response; RCC: renal cell carcinoma; RFI: recurrence-free interval; RFS: relapse-free survival; STS: soft-tissue sarcomas; TAMs: tumor-associated macrophages; T_fh_ cells: t follicular helper cells; T_fr_ cells: follicular regulatory t cells; TNBC: triple-negative breast carcinoma; T_reg_s: regulatory t cells; T_rm_s: resident memory T cells.

**Table 2:** Current methods to detect and quantify TLSs

Methods		Identified parameters	Advantages	Limitations	Ref
Histopathology	H&E/HES staining	Cellular morphology, tissue structure	Low cost, simple operation, widely application	Cannot distinguish specific cells	9, 28, 51, 53, 85, 87
	IHC staining	Specific protein localization/expression	High specificity, precise localization	Limited multiplexing capability, high antibody dependency	44, 72, 76, 82
	IF staining	Multiprotein colocalization (fluorescent labeling)	Multi-color labeling, high resolution (200 nm)	Autofluorescence interference	7, 82, 120, 166
	mIHC/mIF staining	Multiple protein markers	Multi-parametric (4-8 markers)	Antibody cross-reactivity	26, 42, 44, 51, 54, 107
Traditional protein assay methods​	Western blot	Specific protein expression	High specificity, semi-quantitative	Low throughput	167, 168
	ELISA	Soluble protein concentration	High throughput, quantitative accuracy	Single-plex detection, antibody-dependent	23, 167, 169
	Flow cytometry	Cell surface/intracellular molecular markers	Multi-parametric (10^+^ markers), rapid	Limited to cell suspensions, high instrument cost	6, 63, 120, 170
	CyTOF	Metal-tagged proteins	Multi-parametric (40^+^ markers), no spectral overlap	High instrument cost, complex sample preparation	6, 71
Genomics	MSI/MSS analysis	Microsatellite stability	Simple and cost-effective compared to whole-genome sequencing.	Low sensitivity	24, 42, 78
	CGH analysis	Genome copy number variation	Genome-wide coverage, high resolution	Cannot detect balanced translocations or point mutations	82
Transcriptomics	mRNA microarray analysis	Whole transcriptome expression profile	High throughput, moderate cost	Limited to known transcripts, narrow dynamic range	6, 7, 21, 26
	RNA-seq	Whole transcriptome expression profile	Discovery of novel transcripts	High cost, complex data analysis	6, 48, 52, 76
	scRNA-seq	Single cell RNA expression profile	Single-cell resolution	Extremely high cost, technically demanding	6, 7, 44, 47, 171
Spatial transcriptomics	GeoMx DSP	Spatial multi-omics (RNA/ protein)	Preserves spatial context, multi-target analysis	Limited resolution (10-100 μm)	103, 142, 162, 172
	10x Visium	Whole transcriptome spatial localization	Moderate resolution (55 μm, 2μm (HD version)), compatible with FFPE/fresh-frozen tissues	High sample preparation requirements	172, 173
	Stereo-seq​	Subcellular spatial transcriptome	Ultra-high resolution (0.5 μm)	Massive data storage/computational demands	173
Spatial proteomics	IMC	Metal-tagged proteins	Multi-parametric (40^+^ markers), no spectral overlap, compatible with FFPE sections	High instrument cost, complex metal-labeled antibody preparation	47, 174-176
	GeoMx DSP	Spatial multi-omics (RNA/ protein)	Preserves spatial context, multi-target analysis	Limited resolution (10-100 μm)	7, 142, 171, 177
	CyCIF	Fluorescently labeled proteins	High throughput (4-5 cycles, 30^+^ markers), high sensitivity, compatible with standard microscopes	Photobleaching, time-consuming cycles	155
	CODEX	DNA-barcoded proteins	Ultra-high-parametric (50^+^ markers), no spectral unmixing	Complex decoding workflow, antibody validation challenges	178, 179
Radiomics	CT	Anatomical structure, density difference	Rapid, widely application	Radiation exposure, low soft-tissue contrast	139, 180, 181
	SPECT	Blood flow/metabolic distribution	Multi-target imaging	Low resolution, time-consuming	144
	MRI	Soft tissue contrast	No radiation, high resolution	Long scan times, high cost	145
Deep learning model		Automatic image/data feature extraction	Handles complex data, high predictive power	Requires large annotated datasets	140, 149-152
3D imaging technique		3D structure reconstruction	Enables 3D visualization	High equipment cost, complex data processing	141, 154, 155

3D: three-dimensional; CGH: comparative genomic hybridization; CODEX: co-detection by indexing; CT: computed tomography; CyCIF: cyclic immunofluorescence; CyTOF: cytometry by time-of-flight; ELISA: enzyme-linked immunosorbent assay; FFPE: formalin fixed paraffin embedded; GeoMx DSP: GeoMx digital spatial profiler; H&E: hematoxylin and eosin; HES: hematoxylin-eosin-saffron; IHC: immunohistochemistry; IF: immunofluorescence; IMC: imaging mass cytometry; mIHC: multiplex immunohistochemistry; mIF: multiplex immunofluorescence; MRI: magnetic resonance imaging; MSI/MSS: microsatellite instability or stability; PET: positron emission tomography; RNA-seq: RNA sequencing; scRNA-seq: single-cell RNA sequencing; SPECT: single-photon emission computed tomography.

**Table 3:** The prognostic and predictive value of different TLS characteristics in different cancer types

TLS parameters			Cancer types	Cases	Received treatments	Prognostic value	Predictive value to ICB	Ref.
Maturation	Immature TLSs	E-TLSs	CRC	109 patients	Surgical resection; chemotherapy	Association with higher risk of recurrence	NA	42
			LSCC	138 patients	Surgical resection; NAC	Association with shorter PFS, DFS, and OS	NA	74
			PDAC	63 patients	Surgical resection	Association with longer PFS, OS	NA	52
			BC	489 patients	Surgical resection	Association with shorter FS	NA	70
			ESCC	34 patients	ICB (αPD-1); surgical resection	No clinical association	No clinical association	30
			MIBC	153 patients	ICB (αPD-1); NAC	Association with shorter OS	Negative	44
			ccRCC	395 patients	Surgical resection	Association with shorter PFS, OS	NA	51
			UCC	24 patients	ICB (αPD-1 + αCTLA-4); surgical resection	NA	Negative	43
			6 tumor types and others	328 patients	ICB (αPD-1); surgical resection	Association with shorter PFS, OS	Negative	9
	Mature TLSs	PFL-TLSs	HCC	273 patients	Surgical resection	Association with lower risk of recurrence	NA	33
			LSCC	138 patients	Surgical resection; NAC	Association with shorter PFS, DFS, and OS	NA	74
			CRC	109 patients	Surgical resection; chemotherapy	Association with lower risk of recurrence	NA	42
			ESCC	34 patients	ICB (αPD-1); surgical resection	Association with longer PFS	Positive	30
			MIBC	153 patients	ICB (αPD-1); NAC	Association with shorter OS	Negative	44
			UCC	24 patients	ICB (αPD-1 + αCTLA-4); surgical resection	NA	Positive	43
			ccRCC	395 patients	Surgical resection	Association with shorter PFS, OS	NA	51
			6 tumor types and others	328 patients	ICB (αPD-1); surgical resection	Association with shorter PFS, OS	Negative	9
		SFL-TLSs	HCC	273 patients	Surgical resection	Association with lower risk of recurrence	NA	33
			LSCC	138 patients	Surgical resection; NAC	Association with longer PFS, DFS, and OS	NA	74
			CRC	109 patients	Surgical resection; chemotherapy	Association with lower risk of recurrence	NA	42
			PDAC	63 patients	Surgical resection	Association with longer DFS	NA	52
			RCC	59 patients	ICB (αPD-1 + αCTLA-4); surgical resection	Association with longer PFS, OS	Positive	22
			MIBC	153 patients	ICB (αPD-1); NAC	Association with longer OS	Positive	44
			ccRCC	395 patients	Surgical resection	Association with longer PFS, OS	NA	51
			BCa	408 patients	ICB (αPD-1/αCTLA-4/αPD-1 + αCTLA-4); chemotherapy	Association with longer OS	Positive	132
			ESCC	34 patients	ICB (αPD-1); surgical resection	Association with longer PFS	Positive	30
			UCC	24 patients	ICB (αPD-1 + αCTLA-4); surgical resection	NA	Positive	43
			6 tumor types and others	328 patients	ICB (αPD-1); surgical resection	Association with longer PFS, OS	Positive	9
Localization	Intra-tumoral TLSs		NSCLC	74 patients	Surgical resection	Association with longer DSS, DFS, and OS	NA	46
			PDC	534 patients	Surgical resection; chemotherapy; radiotherapy	Association with longer DFS, OS	NA	87
			HCC	273 patients	Surgical resection	Association with lower risk of recurrence	NA	33
			HCC	360 patients	Surgical resection	Association with lower risk of recurrence	NA	79
			CCA	471 patients	ICB (αPD-1); surgical resection; chemotherapy	Association with longer OS	Positive	77
			CRCLM	603 patients	Surgical resection; NAC	Association with longer RFS, OS	NA	26
			GC	53 patients	ICB (αPD-1); surgical resection	Association with longer OS	Positive	67
			ccRCC	395 patients	Surgical resection	Association with longer PFS, OS	NA	51
	Peri-tumoral TLSs		HCC	360 patients	Surgical resection	Association with longer RFS, OS	NA	79
			iCCA	962 patients	Surgical resection	Association with shorter 5-year OS	NA	55
			CCA	471 patients	ICB (αPD-1); surgical resection; chemotherapy	Association with shorter OS	Negative	77
			CRC	174 patients	Surgical resection	Association with longer RFS, OS	NA	78
			CRCLM	603 patients	Surgical resection; NAC	Association with shorter RFS, OS	NA	26
			Cutaneous melanoma	82 patients	Surgical resection; chemotherapy; radiotherapy	Association with longer OS	NA	102
			ccRCC	186 patients	Surgical resection	Association with longer DFS	NA	127
			ccRCC	395 patients	Surgical resection	Association with shorter PFS, OS	NA	51
			GC	53 patients	ICB (αPD-1); surgical resection	Association with longer OS	Positive	67
			ESCC	34 patients	ICB (αPD-1); surgical resection	Association with longer PFS	Positive	30
	Stromal TLSs		HCC	273 patients	Surgical resection	No clinical association	NA	33
			HCC	82 patients	Surgical resection	Association with higher risk of recurrence	NA	82
			GC	53 patients	ICB (αPD-1); surgical resection	Association with shorter OS	Negative	67
			ccRCC	395 patients	Surgical resection	Association with shorter PFS, OS	NA	51
Density			CRC	109 patients	Surgical resection; chemotherapy	Association with lower risk of recurrence	NA	42
			NSCLC	458 patients	Surgical resection	Association with reduced risk of death	NA	85
			NSCLC	147 patients	Surgical resection	Association with longer DFS	NA	71
			LSCC	138 patients	Surgical resection; NAC	Association with longer PFS, DFS, and OS	NA	74
			OSCC	168 patients	NA	Association with longer RFS, 5-year OS	NA	182
			Melanoma	46 patients	ICB (αPD-1/αPD-1 + αCTLA-4); surgical resection	NA	Positive	6
			Melanoma	Mice	ICB (αPD-1/αPD-1 + αCTLA-4)	Association with longer OS	Positive	120
			MIBC	153 patients	ICB (αPD-1); NAC	Association with longer OS	Positive	44
			uLMS	102 patients	Surgical resection	Association with longer OS	NA	72
			ESCC	34 patients	ICB (αPD-1); surgical resection	Association with longer PFS	Positive	30
			PDC	534 patients	Surgical resection; chemotherapy; radiotherapy	Association with longer DFS, OS	NA	87
			UCC	45 patients	ICB (αPD-1 + αCTLA-4); surgical resection	Association with longer RFS, OS	Positive	73
			ccRCC	395 patients	Surgical resection	No clinical association	NA	51

BC: breast carcinoma; BCa: bladder cancer; CRC: colorectal carcinoma; CRCLM: colorectal cancer liver metastases; CTLA-4: cytotoxic T-lymphocyte-associated protein-4; ccRCC: clear cell renal cell carcinoma; DFS: disease-free survival; DSS: disease-specific survival; E-TLSs: early tertiary lymphoid structures; ESCC: esophageal squamous cell carcinoma; GC: gastric carcinoma; HCC: hepatocellular carcinoma; ICB: immune checkpoint blockade; iCCA: intrahepatic cholangiocarcinoma; LSCC: lung squamous cell carcinoma; MIBC: muscle invasive bladder carcinoma; NAC: neoadjuvant chemotherapy; NSCLC: non-small cell lung carcinoma; OS: overall survival; OSCC: oral squamous cell carcinoma; PD-1:programmed cell death protein 1; PDAC: pancreatic ductal adenocarcinoma; PDC: pancreatic ductal carcinoma; PFL-TLSs: primary follicle-like tertiary lymphoid structures; PFS: progression-free survival; RCC: renal cell carcinoma; RFS: relapse-free survival; SFL-TLSs: secondary follicle-like tertiary lymphoid structures; TNBC: triple-negative breast carcinoma; uLMS: uterine leiomyosarcoma; UCC: urothelial carcinoma.

## Abbreviations

3D: three-dimensional
APCs: antigen-presenting cells
ASCs: antibody-secreting cells
AtM: atypical memory
BC: breast cancer
BCa: bladder cancer
B_reg_s: regulatory B cells
CCA: cholangiocarcinoma
CCL: CC motif chemokine ligand
CIN: cervical intraepithelial neoplasia
COAD: colon adenocarcinoma
CRC: colorectal cancer
cSCC: cutaneous squamous cell carcinoma
CT: computed tomography
CTLA-4: cytotoxic T-lymphocyte-associated protein-4
CTLs: cytotoxic T lymphocytes
CXCL: CXC motif chemokine ligand
CXCR: CXC motif chemokine receptor
ccRCC: clear cell renal cell carcinoma
DC-LAMP: dendritic cell-lysosomal-associated membrane protein
DCs: dendritic cells
DFS: disease-free survival
dMMR: defective mismatch repair
DSS: disease-specific survival
E-TLSs: early tertiary lymphoid structures
EF: extrafollicular
ESCC: esophageal squamous cell carcinoma
FDCs: follicular dendritic cells
FRCs: fibroblastic reticular cells
GC: gastric carcinoma
GCs: germinal centers
H&E: hematoxylin and eosin
HCC: hepatocellular carcinoma
HES: hematoxylin-eosin-saffron
HEVs: high endothelial venules
HNSCC: head and neck squamous cell carcinoma
ICI: immune checkpoint inhibitor
ICB: immune checkpoint blockade
IDO1: indoleamine 23-dioxygenase 1
IFN-γ: interferon gamma
IHC: immunohistochemistry
IL-7/IL-7R: interleukin-7/interleukin-7 receptor
ILCs: innate lymphoid cells
iCCA: intrahepatic cholangiocarcinoma
irAEs: immune-related adverse events
LC: lung carcinoma
LED: light-emitting diode
LNs: lymph nodes
LSCC: lung squamous cell carcinoma
LT α1β2/LTβR: lymphotoxin α1β2/lymphotoxin beta receptor
LTi: lymphoid tissue inducer
LTo: lymphoid tissue organizer
MDSCs: myeloid-derived suppressor cells
MHC: major histocompatibility complex
MIBC: muscle-invasive bladder cancer
MRI: magnetic resonance imaging
MSI-H: microsatellite instability-high
mIF: multiplex immunofluorescence
mIHC: multiplex immunohistochemistry
NAC: neoadjuvant chemotherapy
NK: natural killer
NLR: neutrophil-to-lymphocyte ratio
NSCLC: non-small cell lung cancer
OC: ovarian cancer
OS: overall survival
OV: oncolytic virus
oHSV: oncolytic herpes simplex virus-1
PCA: primary cardiac angiosarcoma
PC: pancreatic carcinoma
PCa: prostate cancer
PD-L1: programmed death-ligand 1
PDAC: pancreatic ductal adenocarcinoma
PCs: plasma cells
PET: positron emission tomography
PFL-TLSs: primary follicle-like tertiary lymphoid structures
PFS: progression-free survival
RCC: renal cell carcinoma
SFL-TLSs: secondary follicle-like tertiary lymphoid structures
SLOs: secondary lymphoid organs
SPECT: single-photon emission computed tomography
STS: soft-tissue sarcomas
TAMs: tumor-associated macrophages
TDLNs: tumor-draining lymph nodes
T_fh_: T follicular helper
T_fr_s: follicular regulatory T cells
TGF-β: transforming growth factor beta
Th: T follicular helper
T_h_ 2: T helper 2
TILs: tumor-infiltrating lymphocytes
TLSs: tertiary lymphoid structures
TMB: tumor mutational burden
TME: tumor microenvironment
TNF-α: tumor necrosis factor alpha
T_reg_s: regulatory T cells
T_rm_s: resident memory T cells
uLMS: uterine leiomyosarcoma
UCC: urothelial carcinoma
VSMCs: vascular smooth muscle cells
